# Patient reported outcome measures for visual impairment after stroke: a systematic review

**DOI:** 10.1186/s12955-015-0338-x

**Published:** 2015-09-15

**Authors:** Lauren R. Hepworth, Fiona J. Rowe, Robert Harper, Kathryn Jarvis, Tracey Shipman, Helen Rodgers

**Affiliations:** Department of Health Services Research, Whelan Building (1.10), University of Liverpool, Brownlow Hill, Liverpool, L69 3GB UK; Manchester Royal Eye Hospital, Central Manchester University Hospital NHS Foundation Trust, Manchester, M13 9WL UK; School of Health Sciences, University of Liverpool, Liverpool, L69 3GB UK; Department of Orthoptics, Royal Hallamshire Hospital, Sheffield Teaching Hospitals NHS Foundation Trust, S10 2JF, Sheffield, UK; Institute of Neuroscience and Institute for Ageing, Newcastle University, Newcastle upon Tyne, NE2 4AE UK

## Abstract

**Purpose:**

The aim of this review was to identify patient reported outcome measures (PROMs) for use in research and clinical practice involving individuals with visual impairment following stroke and to evaluate their content validity against quality assessment criteria.

**Method:**

A systematic review of the literature was conducted to identify articles related to the development and/or validation of PROMS. We searched scholarly online resources and hand searched journals. Search terms included MESH terms and alternatives relating to PROMs, visual impairments and quality of life. Data were extracted relating to the development and validation of the included instruments. The quality of the development process was assessed using a modified version of a PROM quality assessment tool.

**Results:**

A total of 142 PROMs were identified, 34 vision-specific PROMs were relevant and available to be analysed in this review. Quality appraisal identified four highly rated instruments: the National Eye Institute Visual Functional Questionnaire (NEI-VFQ), Activity Inventory (AI), Daily Living Tasks Dependant on Vision (DLTV) and Veterans Affairs Low Visual Function Questionnaire (VA LV VFQ). The four instruments have only been used with either a limited number of stroke survivors or a sub-population within visual impairment following stroke.

**Conclusion:**

No instruments were identified which specifically targeted individuals with visual impairment following stroke. Further research is required to identify the items which a population of stroke survivors with visual impairment consider to be of most importance. The validation of a combination of instruments or a new instrument for use with this population is required.

**Electronic supplementary material:**

The online version of this article (doi:10.1186/s12955-015-0338-x) contains supplementary material, which is available to authorized users.

## Introduction

Approximately 152,000 people experience a stroke every year in the UK [1]. The numbers who experience visual problems as a consequence of stroke are not yet accurately known. An estimate of prevalence of post-stroke visual impairment has been reported at around 60 % [2]. There are a wide variety of visual problems which can result from stroke: visual field loss, ocular motility defects, visual inattention, reduced visual acuity and visual perception problems [3–6]. It is possible for a visual problem to occur from a stroke lesion in any area of the brain [7]. Visual impairments have been shown to limit daily activities and independence, contributing to depression and reduced motivation [8, 9].

A patient reported outcome measure (PROM) “addresses some aspect of the patient’s subjective experience of health and the consequences of illness” [10]. These measures can capture an individual’s functionality and feelings related to either their general health or a specific condition. Using self-reporting allows PROMs to capture concepts which would not be possible by any other method [11]. PROMs are used for a wide range of purposes, from establishing the impact of a condition on an individual, assessing the effectiveness of a method of treatment, and as a utility index [12].

Different types of instruments exist, ranging from generic, to disease-specific, to individualised instruments [10]. Generic instruments do not focus on a particular condition, and therefore can be applied to a wide range of population groups as they are broad in scope, e.g. Euro-QoL (EQ-5D), Short Form Health Survey (SF-12) [13, 14]. Disease-specific instruments are tailored to the condition of interest and are more likely to contain items relevant to that disease, e.g. Asthma Quality of Life Questionnaire (AQLQ), Child Amblyopia Treatment Questionnaire (CAT-QoL) [15, 16]. Individualised instruments allow the individual to select the items which are of most importance to them. Firstly, individuals are asked to rank tasks of importance to their lives, then subsequently the effect of their health condition on those specific tasks. e.g. Patient Generated Index (PGI), Schedule for the Evaluation of Individual Quality of Life (SEIQoL) [17, 18]. It is possible for PROMs to cover more than one of these types.

Literature reviews of PROMs for ocular conditions causing visual impairment, such as glaucoma and cataract, have been conducted, some of which give recommendations for which instrument to use for different disease specific populations [19–22].

NHS services are increasingly being asked to provide evidence of the impact of their care [23]. One method of achieving this objective is by asking individuals to complete a PROM. For example, since 2009, data has been collected nationally in England, before and after four commonly performed elective surgical procedures (hip replacements, knee replacements, varicose vein and groin hernia surgery) using PROMs [12].

The aim of this review was to identify PROMs available for use in research and clinical practice involving individuals with visual impairment following stroke, and to evaluate their content validity against quality assessment criteria. This review will focus on high quality instruments which have previously been validated with stroke survivors. A secondary aim is to highlight suitable high quality alternative instruments which have not yet been validated for stroke survivors.

## Methods

### Search strategy

A systematic search strategy was used to search the following key electronic databases: MEDLINE (1948 to August 2014), SCOPUS (1823 to August 2014), AMED (1985 to August 2014), CINAHL (1937 to August 2014) and PsycINFO (1887 to August 2014). Citation tracking was performed using Web of Science Cited Reference Search for all included studies, and reference lists of included articles were searched. Search terms included a variety of MESH terms and alternatives in relation to patient reported outcome measures, visual impairments and quality of life (Table 1).

**Table 1:**
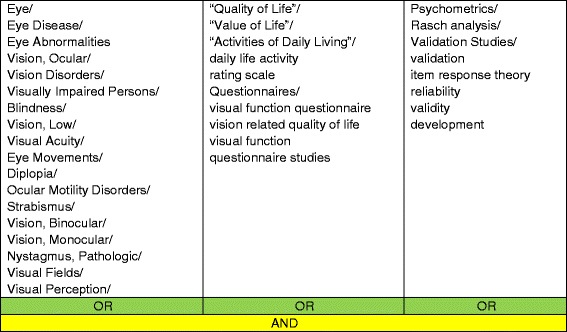
Search terms

### Inclusion and exclusion criteria

Articles related to the development and/or validation of PROMs for adult stroke survivors were included. Articles related to ocular stroke (central and branch retinal artery occlusion) were deemed to be outside the scope of this review and were therefore excluded. Some of the visual problems experienced following a stroke are also experienced by other population groups, for example visual field loss is also experienced with glaucoma and blurred reduced vision is experienced with cataracts. Therefore, articles related to the development and/or validation of PROMs for individuals with visual impairment which could be experienced following other ocular conditions were also included. Studies evaluating questionnaires in languages other than English were excluded, unless the questionnaire was originally developed in another language and later translated to English. PROMs which were not accessible, for example if they required payment to view, were excluded.

### Selection of studies

The titles and abstracts identified from the search were screened using the pre-stated inclusion criteria. The full papers of any studies considered potentially relevant were then considered and the selection criteria applied.

### Quality assessment

All included PROMs were quality assessed using a modified version of a published quality assessment tool [20, 24]. The modified quality assessment tool is shown in Table 2. The tool is made up of two parts, the first evaluates the development of the instrument and the second evaluates the performance of the instrument in terms of validity and reliability [24]. For the purposes of this review, six items from part one were relevant, focusing on evaluating the development of the instrument. For each of the quality assessment items the instruments were judged against specific criteria to have a positive rating (**√√**), a minimal acceptable rating (√) or a negative rating (X), and if information relating to the criteria was not reported ‘NR’ was recorded [24]. The quality assessment focused on the robustness of the methodology of development rather than the reliability and validity of the instruments. The validity in the target population does not necessarily translate to the instrument being valid or appropriate to a stroke population.

**Table 2:** Quality assessment tool for evaluation of PROMs modified from Pesudovs et al. [24] and Hamzah et al. [20]

Quality criteria	Definition	Quality criteria
Pre-study hypothesis	The pre-study specification of the aim of the instrument and the intended population	**√√** A clear description is provided of the aim of the instrument and the intended population
		**√** Only one of the above
		**X** Neither reported
Intended population	The extent to which the instrument has been studied in the intended population	**√√** Intended population studied
		**√** Partly studied only or sample size was small (less than 50 patients)
		**X** Not studied in the intended population, only generic
Actual content area	The extent to which the content meets the pre-study hypothesis specifications	**√√** Content is intended and is relevant to the intended population
		**√** Some of the intended content areas are missing
		**X** Content areas are not relevant to the intended population
Item identification	Selection of the items relevant to the target population for inclusion in the pilot instrument	**√√** Comprehensive consulting with patients (focus groups or in-depth interviews) and a literature review
		**√** Minimal consultation with patients and expert opinion and literature review
		**X** No consultation with patients
Item selection	Determining the items included in the final instrument	**√√** A pilot instrument was developed and tested with Rasch or factor analysis and statistical justification provided for removing items, plus items with floor and ceiling effects removed and the amount of missing data considered
		**√** Only one of the above techniques were used
		**X** No pilot instrument OR no statistical justification of items included in the final instrument
Scoring	A description of how the instrument should be scored	**√√** Rasch scoring of a statistically justified response scale
		**√** Summary scoring of a statistically justified response scale
		**X** Scoring system not described or scoring of a statistically unjustified or faulty scale

If not reported, scored as ‘NR’; **√√** positive rating; **√** minimal acceptable rating; **X** negative rating

### Data synthesis

A descriptive analysis table (Additional file 1) was completed from the included articles with the following data: initial aim of the PROM, the intended population, how items were identified, whether stroke survivors were part of the development process, the process for selecting items included in the instrument and the scale, the validation processes including populations for which the instrument has been validated. The quality assessment data was synthesised using a graphical representation for each ratings into a table (Table 3).

**Table 3:**
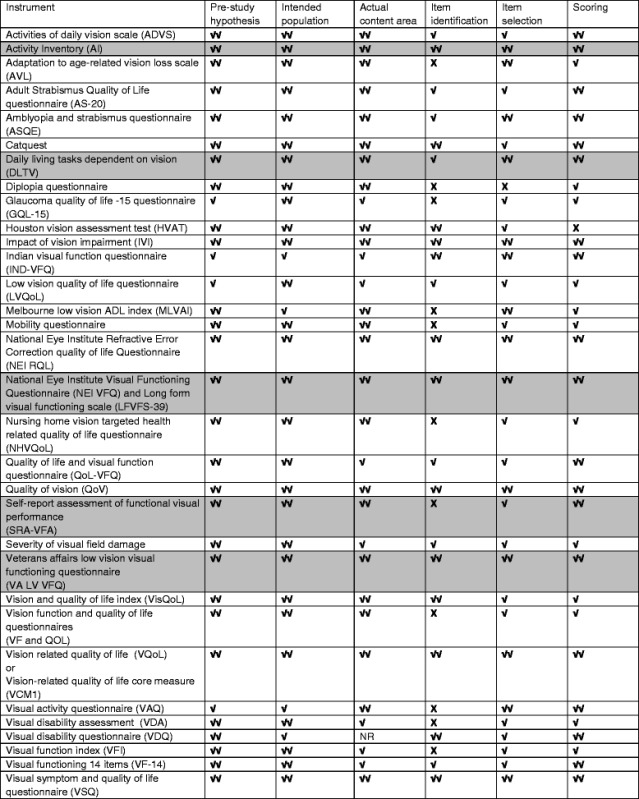
Results of the quality appraisal of the content validity of the included instruments

If not reported, scored as ‘NR’; **√√** positive rating; **√** minimal acceptable rating; **X** negative rating

Gray color: Used with stroke patients

## Results

The search revealed 142 PROMs of which 43 vision-specific PROM instruments were identified as being relevant. However, nine of these instruments were excluded as they were not accessible. Lack of accessibility was due to requiring payment or no development or validation papers could be found for an instrument. A total of 34 vision-specific PROMs were analysed for this review. Specific details of all PROMs included are shown in Additional file 1.

### Target condition

None of the instruments reviewed had been specifically targeted at visual impairment following stroke. Eighteen of the instruments were developed for populations with visual impairment with no specific condition targeted. As this group of instruments were aimed generally at visual impairment, it was difficult to establish if stroke survivors were included in the populations recruited by studies reporting the use of these instruments. Of the remaining instruments, eight were cataract-specific, three were strabismus/amblyopia-specific, two were glaucoma-specific, two were retinal disease-specific and one was refractive error-specific.

None of the PROMs included in the review sought the views of stroke survivors during the item identification process. The Neuro-10 supplement was created to adapt the National Eye Institute Visual Functional Questionnaire (NEI-VFQ) to be better targeted to a population experiencing visual impairment due to neuro-ophthalmic disorders [25]. Of note, however, is the item identification process of the Neuro-10 supplement only involved individuals with multiple sclerosis.

### Administration

The methods of administration varied between interview, self-administration and a combination of both. Details of the administration methods used by each instrument are outlined in Additional file 1. A study into the most appropriate method of administration of vision-related quality of life instruments concluded postal administration to be the most reliable, valid and cost-effective [26]. However, depending on the severity of visual impairment it may not be possible for an individual to complete a self-administration of an instrument [27]. It is important to consider the method which best suits the population group and/or the individual [28].

### Instrument content

The instruments had a broad range in the number of items per instrument, the smallest being the Vision and Quality of Life Index (VisQoL) with six items, and the largest being the Activity Inventory (AI) with up to 337 items [29, 30]. The mean number of items was 39 (SD 57.7) across the instruments reviewed and the median number of items was 25 (IQR 17 to 38).

### Instrument development and quality

#### Instruments validated with stroke survivors

Content validity assesses if the instrument and individual items are relevant to the target population and are able to measure the area of interest [31]. A summary of the descriptive analysis of the development and content validity for each instrument is provided in Additional file 1 and the quality assessment is available in Table 3. Five instruments were found to have been tested with stroke survivors: NEI-VFQ (Neuro 10), AI, DLTV, (Daily Living Tasks Dependant on Vision), VA LV VFQ (Veteran Affairs Low Visual Function Questionnaire) and SRA-FVP (Self-Reported Assessment of Functional Visual Performance). In order to focus on the aim, the remainder of this review will concentrate only on the analysis of these instruments [25, 30, 32–35].

The instrument found to have the highest number of positive ratings in the quality assessment was the NEI-VFQ 25 (Neuro 10). The NEI-VFQ 25 is composed of 11 vision-related subscales: vision rating, near vision activities, distance vision activities, social functioning, role limitation, dependency, mental health, driving, peripheral vision, colour vision and ocular pain with an additional question for general health rating [32]. There is also the option to add items to a specific sub-scale. The instrument provides an overall composite score [36]. It is unclear if any stroke survivors were involved in the item identification of the NEI-VFQ 25 or Neuro 10 supplement as the population had a variety of causes of visual impairment including neurological aetiologies. Five studies (*n* = 608) have subsequently used this instrument to assess quality of life in individuals with visual impairment following stroke, especially in individuals with homonymous hemianopia [9, 37].

Of the instruments previously used with a stroke population two were ranked as joint second with regard to quality assessment. These were the AI and the VA LV VFQ. During the development stages of these instruments, stroke survivors were not involved in item identification.

The validation process for the AI involved a population with visual impairment due to a variety of aetiologies. This population included a small proportion (3 %) with stroke or traumatic brain injury [38]. The AI uses a theoretical framework called Activity Breakdown Structure to allow the questionnaire to be adapted for each individual. At the highest level of this structure are three ‘objectives’: daily living, social interaction and recreation [30]. Under these headings are 41 ‘goals’, for example cooking a meal, which would be required to achieve the ‘objective’ of daily living. The ‘goals’ are then divided into the specific ‘tasks’ of which there are 337, for example reading a recipe, measuring ingredients and reading oven dials, which must be achieved to successfully complete the ‘goal’. The importance of each ‘goal’ is initially rated by the individual, and if it is not considered important, the next ‘goal’ is considered. If it is deemed important the individual is asked to rate the difficulty of the ‘tasks’ that make up that ‘goal’ [30]. The design of this instrument allows the number of items to vary depending on the number of goals important to the individual.

The VA LV VFQ was originally validated with patients with ophthalmic pathology such as glaucoma, macular degeneration and diabetic retinopathy [34, 39, 40]. It was later used with a small group (*n* = 24) of stroke survivors with homonymous hemianopia [41]. The VA LV VFQ is composed of five domains: visual ability, reading, mobility, visual motor and visual information. The instrument consists of a total of 48 items, with each item made up of four questions: for example: “Is it difficult to read menus?”. If the answer to the first question is yes, the following questions are subsequently asked “Is it because of your vision?”, “Do you want training to read menus?” and finally “How do you usually read menus?” [40].

The instrument ranked next with regard to quality assessment was the DLTV. The DLTV was originally developed for use with individuals with macular degeneration. It was later used with a group of stroke survivors with visual impairment, the total population was large (*n* = 915), however, only 63 participants were reported to have completed the questionnaire [42]. It comprises 24 items which are not categorised under named domains, but covers topics such as reading, mobility, self-care and recognition [35]. Fifteen of the items use the following question “How much difficulty do you have pouring yourself a drink”. Two mobility questions use “How confident are you in your ability to walk around in your immediate neighbourhood”. Five questions on reading use the following question “With your near glasses on, how much difficulty do you have reading normal sized newspaper print”. The final two questions ask “How would you rate your overall distance and near vision” [35].

The other instrument which has previously been used with stroke survivors is the SRA-VFP, however this use was limited to individuals with homonymous hemianopia [33]. This instrument consists of 38 items covering a range of activities of daily living: reading, clothing care, meal preparation, leisure participation, financial management, shopping, writing, communication, health management, social participation, functional mobility, personal hygiene, feeding and dressing. The individual completing the instrument is ask to rate their ability to perform each task. This instrument scored a lower rating on quality assessment than the NEI-VFQ (Neuro 10), AI, VA LV VFQ and DLTV, as patients were not consulted in the item selection process it only involved expert opinion.

#### Instruments not yet validated with stroke survivors

Of the other instruments not previously tested with stroke survivor populations, a number achieved high positive ratings and might be appropriate for use with a specific visual condition or symptom arising due to stroke. For instance the Diplopia questionnaire or the Adult Strabismus Quality of Life questionnaire (AS-20) could be used with stroke survivors experiencing ocular motility problems [43, 44]. None of the high positive rating instruments in the quality assessment were found to be specific for visual field loss. The instruments for specific visual conditions or symptoms (Diplopia questionnaire and AS-20) are unlikely to be suitable for use with stroke populations experiencing varied and mixed visual impairment post-stroke.

The AS-20 is comprised of 20 items originally divided equally into two domains psychological and function. The domains were later divided further into self-perception, interactions, reading function and general function [43, 45]. The questions are statements which the individual is asked to record the frequency of occurrence.

The Diplopia Questionnaire consists of eight items, the first question is a filter question asking if diplopia has been noticed in the past week. If yes, the following items record the frequency of diplopia in seven positions of gaze, simply asking if “During the last week, did you have double vision when reading (in a normal reading position” [44].

An alternative to using these instruments for specific visual conditions is to use a vision-specific instrument which has no target condition. Four such instruments achieved the highest positive rating in quality assessment: Impact of Vision Impairment (IVI), Quality of Vision (QoV), Vision Related Quality of Life (VQoL) and Visual Symptom and Quality of Life Questionnaire (VSQ) [46–49].

The IVI consists of 28 items within six domains: emotional reaction to vision loss, household care, personal care, leisure and work, mobility and social and consumer interactions. The question focuses on the last month and the frequency of impairment, for example “In the past month, how much has your eyesight interfered with visiting friends or family” [50].

The QoV is made up of 30 items covering ten symptoms: glare, haloes, starbusts, hazy vision, blurred vision, distortion, double vision, fluctuation, focusing difficulties, and judging distances. The questions ask regarding the frequency, severity and bothersomeness of each symptom [49].

The VQoL is a parent questionnaire which can contain up to 139 items, this instrument has a modular approach to enable it to meet the requirements of different population groups. The questions focus on the past month, for example “In the past month, how much has your eyesight interfered with seeing food on the plate” or “In the past month, how often have you felt anxiety because of your eyesight” [48]. A core set of ten items were identified which became the VCM1. All items within the VCM1 relate to emotional feelings and concerns, such as embarrassment, frustration and worry [51]. There is no method reported on how to decide if additional items are required, but it is left flexible for the clinician or researcher to decide dependant on the individual completing the questionnaire.

The VSQ has the option of either a long or a short version. The long version consists of 26 items and the short form is made up of 14 items, across two domains symptoms/dysfunction and vision-specific quality of life. There is no standard question wording, but examples include “When you are watching television, do you find it difficult to see the picture clearly” and “How often does your eyesight prevent you from doing the things you would like to do” [46].

The IVI, VQoL and VSQ have been validated for use with many different types of visual impairment, and therefore may be suitable for use with a whole stroke population [46, 48, 50–59].

## Discussion

This review quality appraised existing vision-specific PROMs to identify those which could be used for individuals with visual impairment following stroke. All instruments included in the review could potentially be relevant for use when assessing the impact of visual impairment following stroke, due to the wide variety of visual problems which may occur as a result of a stroke. No instruments were clearly identified as involving stroke survivors in item identification. As a consequence none of the currently available instruments have been influenced, during their development, by stroke survivors. This limitation potentially results in instruments having irrelevant items or not containing pertinent items for the stroke population. Five instruments (AI, NEI-VFQ, VA LV VFQ, DLTV and SRA-VFP) have been administered with a stroke population embedded within larger mixed population studies or a relatively small sample [30, 32, 33, 41, 42]. The SRA-VFP is not discussed in detail in this review.

A stroke population has a wide variety of potential visual defects, for example ocular motility defects and visual perception defects. This instrument has not, to our knowledge, been validated for the wider stroke population. The NEI VFQ (Neuro 10) and VA LV VFQ, have been used with stroke survivors, this use was restricted to a subpopulation and if the instrument is to be used with stroke survivors with all forms of visual impairment, it requires further validation.

There are also problems with question phasing in the AI and DLTV, they do not include a reference to vision or eyesight, but simply “how difficult is it for you to walk without assistance of another person?” or “how much difficulty do you have cutting up food on your plate” respectively [38, 60]. Stroke survivors commonly have other new physical and cognitive deficits in addition to visual impairment. It would not be clear from the AI which deficit (visual/physical/cognitive) was causing, either fully or partially, the difficulty experienced. Considering the limitation of these instruments it is advisable that they should not be used for assessment of vision-specific quality of life with stroke populations with visual impairment.

The VA LV VFQ has the potential to include up to 192 questions depending on the number of goals the individual judges to be important, this is a high number considering the individual completing the instrument has visual impairment and potentially cognitive impairment [40]. It is also well documented that the stroke population are prone to fatigue [61]. Taking these factors into account this instrument would not be the best fit for a stroke population. The DLTV could be regarded as being more suitable for completion by patients with regard to the fewer number of items. This instrument has previously been used with a population of stroke survivors who had a wide range of visual impairments [62].

Some alternative instruments were identified during the review. These were vision-specific instruments with no target condition (IVI, QoV, VQoL and VSQ) with the potential for use with stroke populations and other instruments for specific visual impairments (AS-20 and Diplopia questionnaire) following stroke. The IVI has question phasing limitations similar to that of the AI and DLTV, however, in this case some distinction can be made at the extreme end of scoring ‘can’t do because of eyesight’ ‘can’t do because of other reasons’. In cases where the participant can do the activity but has a degree of difficulty, it is not possible to identify if this is due to visual impairment or other reasons. For this reason this instrument would not be recommended for use with a stroke population with visual impairment.

For the sub-population of stroke patients with ocular motility defects, the AS-20 would be recommended over the Diplopia questionnaire. The AS-20 assesses quality of life, whereas, the Diplopia questionnaire establishes the presence or absence of diplopia in different positions of gaze.

It is important to acknowledge that none of these instruments have previously been validated for use within a stroke population. The vision-specific instruments without a target condition were of higher ranking in the quality assessment than the specific visual impairment instruments. If these were to be used for assessing vision-related quality of life in a stroke population, further validation is recommended.

The quality assessment tool could be viewed as being biased towards the use of Rasch analysis. However, there has been a steady increase in the use of the Rasch model within vision-specific quality of life measures over the years. Many instruments which were conventionally developed have been re-engineered using the Rasch model [63]. This is supported by 76 % of the instruments included in this review have been evaluated using Rasch analysis. Although conventional methods are still popular and summary scoring viewed as straightforward, Rasch offers a robust measure of internal construct validity and takes into account item difficulty by transforming the ordinal scale to an interval scale [64].

## Conclusion

In this review, no instruments were developed specifically for visual impairment following stroke or involved stroke survivors in the item identification phase of instrument development. Five instruments have subsequently been used with stroke survivors. Four of these instruments (AI, NEI-VFQ, DLTV and VA LV VFQ) scored highly on positive ratings in the quality appraisal. Three are vision-specific questionnaires and intended for a broad population of individuals with visual impairment. The exception is the DLTV which was originally developed for individuals with macular degeneration. Other instruments (IVI, QoV, VQoL and VSQ) were identified in this review as having a potential application with stroke survivors with visual impairment. Of the instruments highlighted it is difficult without formal testing to recommend which would be most appropriate for use with a stroke population. However, the following instruments, AI, DLTV, VA LV VFQ and IVI have been highlighted as not be suitable for a stroke population due to question phasing and response burden.

A combination of instruments may be required to cover areas relevant to specific forms of visual impairment which are important for the population of stroke survivors with visual impairment. Further research is required to (a) consult a stroke population with different forms of visual impairment with regard to the items that they judge to be important and (b) to develop or validate appropriate instruments for use with this population.

## Additional file

Additional file 1:
**Descriptive analysis of PROMs.** (DOCX 167 kb)

## Abbreviations

AI: Activity Inventory
AQLQ: Asthma Quality of Life Questionnaire
CAT-QoL: Child Amblyopia Treatment Questionnaire
DLTV: Daily Living Tasks Dependant on Vision
EQ-5D: Euro-QoL
IVI: Impact of Visual Impairment
NEI-VFQ: National Eye Institute Visual Functional Questionnaire
PGI: Patient Generated Index
PROM: Patient reported outcome measure
SEIQoL: Schedule for the Evaluation of Individual Quality of Life
SF-12: Short Form Health Survey
SRA-VFP: Self-Reported Assessment of Functional Visual Performance
VA LV VFQ: Veterans Affairs Low Vision Visual Function Questionnaire
VisQoL: Vision and Quality of Life Index
VQoL: Vision related Quality of Life
VSQ: Visual Symptom and Quality of Life Questionnaire
