# Supplementary material for: Patient reported outcome measures for visual impairment after stroke: a systematic review
Source: Health Qual Life Outcomes. 2015 Sep 15;13:146. doi: 10.1186/s12955-015-0338-x (PMC4572686; doi:10.1186/s12955-015-0338-x)
Supplement: Additional file 1: — Descriptive analysis of PROMs. (DOCX 167 kb) [file 12955_2015_338_MOESM1_ESM.docx]

| **Additional File 1: Descriptive analysis of patient-reported outcome measures** | | | | | | | | | |
| --- | --- | --- | --- | --- | --- | --- | --- | --- | --- |
| Instrument | Aim | Intended population | Item identification | Views of stroke patients considered | Item selection | Population instrument was validated | Actual content area | Scale | Method of administration |
| Activities of daily vision scale (ADVS) | To assess visual function in patients with cataracts to assess timing for surgery for use in research and clinical settings. | Patients with cataracts | *Literature review/other instruments:* No  *Lay focus group:* 15 cataract patients  *Expert focus group:* No  *Expert opinion:* 6 Ophthalmologists (Mangione et al., 1992) | No | A pilot instrument was developed and questions were eliminated from the draft questionnaire on the basis of factor analysis (Mangione et al., 1992, Valbuena et al., 1999), Rasch analysis reduced to 15 items with a compromise between good item fit and precision (Pesudovs et al., 2003). Further Rasch analysis reduced to 8-items predominantly from the near vision scale (ADVS-Near Vision Scale) (Gothwal et al., 2010b) | Patients scheduled for cataract extraction  (Mangione et al., 1992, Pesudovs et al., 2003, Gothwal et al., 2010b)  Elderly community (Valbuena et al., 1999) | Distance vision activities  Near vision activities  Glare disability  Night and daytime driving | Ordinal 5 point scale – 1 =‘Do not do the activity because of vision problems’ to 5 = ‘Not difficult at all’  Reduced to a 4-point scale  Combining points 2 and 3 to create ‘a lot of difficulty’ (Massof, 2007, Gothwal et al., 2010b) | Interview (Valbuena et al., 1999, Massof, 2007)  Self-administration (Gothwal et al., 2010b) |
| Activity Inventory (AI) | Assess functional history and visual ability | Patients with visual impairment | *Literature review/other instruments:* Yes  *Lay focus group:* No, 30-45 minute interviews conducted with 3200 patients  *Expert focus group:* No  *Expert opinion:* Yes, 17 AAO diplomats in low vision were surveyed | No | A pilot questionnaire (3 objectives, 24 goals and >200 activities) was developed using the Activity Breakdown Structure (Massof, 1998). During testing patients were able to suggest additional activities relevant to them which were not included. Modification following pilot study resulted in the tool containing 41 goals and 337 activities (Massof et al., 2005a). Further Rasch analysis made no changes to included items (Ahmadian and Massof, 2008) | Patients attending the low vision service (ARMD, glaucoma, diabetic retinopathy, refractive disorders, cataract, corneal disorders and other conditions causing visual impairment) (Massof et al., 2005a). In addition to the above listed conditions (stroke, brain injury and developmental disorders) (Massof et al., 2005b), (ocular injuries and retinal detachments (Massof et al., 2007). | Daily living  Social interaction  Recreational activities (Massof, 1998)  Alternative subcategories:  Visual information  Mobility  Reading  Visual motor  (Massof et al., 2005b) | Ordinal 6 point scale – 0 = ‘not important’ to 5 = ‘extremely important’ and Ordinal 6 point scale – 0 = ‘not difficult’ to 5 = ‘extremely difficult or impossible’ (Massof, 1998)  Following Rasch analysis categories 1 and 2 and 3 and 4 were collapsed to create a 4 point scale – 0 = ‘not difficult’ to 3 = ‘impossible’ (Massof et al., 2005a, Massof et al., 2005b) | Interview (Massof et al., 2005a, Massof et al., 2005b, Massof et al., 2007) |
| Adaptation to age-related vision loss scale (AVL) | To assess the psychological adaptation of older people to visual impairment | Older patients with visual impairment | *Literature review/other instruments:* Yes  *Lay focus group:* No  *Expert focus group:* No  *Expert opinion:* No | Unclear | A pilot 33-item questionnaire was developed, through initial testing the number of items reduced to 24 (Horowitz and Reinhardt, 1998). The number of items was reduced again to develop a short-form of the questionnaire. The decisions to remove 10 items were based on skewness, interviewer feedback, missing data and low correlations. A further 2 items were removed through factor analysis resulting in the AVL-12 (Horowitz et al., 2007). Rasch analysis was performed on the original AVL-24, in order to achieve unidimensionality 5 items were deleted and a further 3 misfitting items were removed (Gothwal et al., 2009a). | Patients ages >65 years with visual impairment (Horowitz and Reinhardt, 1998). Older patients with visual impairment (glaucoma, cataract, ARMD and diabetic retinopathy) (Horowitz et al., 2007). Patients with cataracts (Gothwal et al., 2009a) | Acceptance of the vision loss  Attitudes towards rehabilitative training  Attitudes towards relationships with family members and friends | Agree or disagree (Horowitz and Reinhardt, 1998)  Ordinal 4-point scale – 3 = ‘strongly agree’ to 0 = ‘disagree’ (Horowitz et al., 2007) | Interview (Horowitz and Reinhardt, 1998) |
| Adult Strabismus Quality of Life questionnaire (AS-20) | Evaluate health related quality of life in adults with strabismus | Adults with strabismus | *Literature review/other instruments:* No  *Lay focus group:* No – 30 individual interview with patients  *Expert focus group:* No  *Expert opinion:* No | Unclear | The draft questionnaire developed contained 181 items from unique statements from the patient interviews. This was reduced to 20 items using factor analysis (Hatt et al., 2009). Rasch analysis reduced the questionnaire by two items (hobbies and depth perception) to 18 items (Leske et al., 2012) | Patients with childhood and acquired strabismus (Hatt et al., 2009). | Psychosocial  Function  Subscales were divided following Rasch analysis:  Self-perception  Interactions  Reading function  General function  (Leske et al., 2012) | Ordinal 5-point scale – 100 = ‘never’ to 0 = ‘always’ plus a ‘not applicable’ option  Rasch analysis indicated to collapse never and rarely in the function subscale (Leske et al., 2012) | Self-administration (Hatt et al., 2009, Leske et al., 2012) |
| Amblyopia and strabismus questionnaire (ASQE) | Evaluate health related quality of life in patients with amblyopia and strabismus | Adults with strabismus | *Literature review/other instruments:* Existing questionnaires  *Lay focus group:* Yes  *Expert focus group:* No  *Expert opinion:* No | No | A pilot questionnaire was developed using themes from the patient focus group (van de Graaf et al., 2004). Translation of Dutch version (26 items) to English (Felius et al., 2007).  Rasch analysis highlighted problems with testing non-strabismic amblyopes, a reduction of 3 items was required to improve fit (Vianya-Estopa et al., 2010) | Patients with strabismus with or without amblyopia (van de Graaf et al., 2004, Felius et al., 2007, Vianya-Estopa et al., 2010) | Fear of losing the better eye  Distance estimation  Visual disorientation  Double vision  Social contact and appearance | Ordinal 5-point scale – 1 = ‘None of the time’ to 5 = ‘All of the time’ (van de Graaf et al., 2004)  Rasch analysis suggested the collapse of categories 4 (some of the time) and 5 (all of the time) (Vianya-Estopa et al., 2010) | Self-administration (Felius et al., 2007, Vianya-Estopa et al., 2010) |
| Catquest | Assess benefits of cataract surgery | Patients before and after cataract surgery | *Literature review/other instruments:* Yes  *Lay focus group:* No, 139 patients were interviewed  *Expert focus group:* No  *Expert opinion:* No | No | The questionnaire started with 37 items, this was reduced using results from patient interviews to 6 activities which were deemed to be important plus a question about the persons preferred activity, creating an 18 item questionnaire. (Lundstrom et al., 1994). Rasch analysis suggested the removal of frequency and symptom items. The remaining disability and global items were combined to create a 9-item short form measure (Catquest-9SF) (Lundstrom and Pesudovs, 2009). Another Rasch analysis agreed with the removal of the frequency items, however, also kept two symptom items and one driving item – creating a 12 item questionnaire (Gothwal et al., 2009b). | Patients awaiting cataract surgery (Lundstrom et al., 1997, Lundstrom and Pesudovs, 2009) | Frequency of performing activities  Perceived difficulty in performing activities  Difficulties in general and satisfaction with vision  Cataract symptoms | Ordinal 5-point scale – 1 = ‘No difficulty’ to 5 = ‘Cannot perform the activity because of bad vision’ plus a ‘cannot say’ option (Lundstrom et al., 1997)  Following Rasch analysis, categories 3 and 4 in the frequency scale were collapsed (Lundstrom and Pesudovs, 2009). | Interview (Lundstrom et al., 1997) |
| Daily living tasks dependent on vision (DLTV) | Evaluate an individual’s visual status | Patients with age-related macular degeneration | *Literature review/other instruments:* No  *Lay focus group:* Yes – list activities which cause difficulties  *Expert focus group:* No  *Expert opinion:* Yes – asked for comment on patients lists | No | A pilot question was developed using the activities suggested by patients and health care professionals, it consisted of 22 items plus 2 overall visual function rating scales (Hart et al., 1999).  Item response theory analysis found two redundant items (reading correspondence and identifying money), therefore these were removed (Hart et al., 2005).  Rasch analysis was performed on the original 22 item questionnaire, resulted in the removal of 5 items (adjusting to dark, objects off to one side, seeing and using steps and pouring a drink) reducing the overall questionnaire to 17 items (Denny et al., 2007) | Patients with ARMD or awaiting cataract surgery with a control group from an elderly population (Hart et al., 1999). Patients with ARMD (Hart et al., 2005, Schmier et al., 2006).  Patients with visual impairment following stroke (Rowe et al., 2013) | The domains are not named (Hart et al., 2005) | Ordinal 4-point scale – 1 = ‘Cannot see to do’ to 4 = ‘No difficulty’ (Hart et al., 1999)  Rasch adjusted 4-point scale was found to be optimal (Denny et al., 2007) | Interview (Hart et al., 2005)  Self-administration (Schmier et al., 2006) |
| Diplopia questionnaire | To assess the frequency of diplopia | Patients with diplopia | *Literature review/other instruments:* No  *Lay focus group:* No  *Expert focus group:* No  *Expert opinion:* Yes | Unclear | A short questionnaire was developed with 8 items, one for each position of gaze (Holmes et al., 2005).  The questionnaire was revised, which resulted in the removal of one item (Holmes et al., 2013) | Patients with acquired diplopia (Holmes et al., 2005) | - | Ordinal 3-point scale – ‘always’ to ‘never’, weighted scores to primary and reading position (Holmes et al., 2005)  ‘Rarely’ and ‘Often’ were added creating a 5-point scale (Holmes et al., 2013) | Interview (Holmes et al., 2005) |
| Glaucoma quality of life -15 questionnaire  (GQL-15) | Assess the impact of common disabilities suffered by patients with glaucoma | Patients with glaucoma | *Literature review/other instruments:* Yes – existing questionnaires for glaucoma and other visual conditions  *Lay focus group:* No  *Expert focus group:* No  *Expert opinion:* No | No | A pilot questionnaire underwent item reduction to 15 items using factor analysis (Nelson et al., 1999).  The GQL-15 was assessed using Rasch analysis. Items were removed to eliminate multidimensionality (reading newspaper, tripping, crossing the road, bumping into objects, recognising faces and adjusting to bright lights). The re-engineered instrument was named the Glaucoma Activity Limitation-9 (GAL-9)(Khadka et al., 2011).  A second Rasch analysis of the GQL-15 was completed and Items were reduced to 10 and renamed Glaucoma Activity Limitation-10 (GAL-10) (Gothwal et al., 2012b).  A more recent Rasch analysis found 2 misfit items, however, removal did not improve fit statistics and concluded the original GQL-15 is valid (Wang et al., 2013) | Patients with glaucoma | Outdoor mobility and navigation  Lighting and glare  Activities demanding functional peripheral vision  Household tasks and personal care | Ordinal 5 point scale – 1 = ‘no difficulty’ to 5 = ‘severe difficulty’ plus a ‘not relevant’ option (Khadka et al., 2011). | Self-administration (Nelson et al., 1999)    Combination of self-administration and interview (Gothwal et al., 2012b)  Interview (Wang et al., 2013) |
| Houston vision assessment test (HVAT) | Aid the decision making process when considering cataract surgery | Patients with cataracts | *Literature review/other instruments:* No  *Lay focus group:* Yes  *Expert focus group:* No  *Expert opinion:* No | No | A pilot questionnaire was developed containing 11 items. For each item both physical and visual impairment is estimated (Prager et al., 2000).  Rasch analysis was not conducted as the rating scale could not be fixed (Gothwal et al., 2011b) | Patients awaiting cataract surgery (Prager et al., 2000) | Cooking  Night driving  Day driving  Housework  Leisure activities  Outdoor activities  Reading  Taking medication  Watching television  Writing | Part A - Ordinal 5 point scale – 0 = ‘not at all limited’ to 4 = ‘severely limited’ plus a ‘not relevant’ option.  Part B - Ordinal 6 point scale – 0 = ‘I have no visual or other physical limitations’ to 5 = ‘all due to eyesight’ (Prager et al., 2000)  Rasch analysis of scales  Part B – reduced to a 4-point scale 0 = ‘none due to eyesight’ to 3 = ‘all due to eyesight’, categories 2 and 3 were collapsed.  Reanalysed indicated more adjustment to the scales were required but it could not be isolated which categories needed to be collapsed (Gothwal et al., 2011b) | Self-administration (Gothwal et al., 2011b) |
| Impact of vision impairment (IVI) | To assess the impact of vision impairment on a visually impaired patient’s ability to participate in daily life activities | Patients with various visual impairments | *Literature review/other instruments:* Existing QoL questionnaires, VF-14, NEI-VFQ, VQOL and VCM1.  *Lay focus group:* 53 patients with visual impairment (diabetic retinopathy, glaucoma, ARMD, cataracts, retinitis pigmentosa and congenital visual impairments)  *Expert focus group:* No  *Expert opinion:* No  (Keeffe et al., 1998) | No | A draft questionnaire (76 items) based on a combination of the VQOL and VCM1, excluding items on ocular symptoms and disability, with the addition of focus group suggestions. A reduction in items to 45 due to high correlation (Keeffe et al., 1999). A further 13 items were excluded due to redundancy (items in emotional domain) and floor and ceiling effects (eating and driving); factor analysis was unable to highlight items for exclusion (Hassell et al., 2000). The items were reduced further by Rasch analysis to 28 due to irrelevance (work and sporting events) and ceiling effects (hobbies and reading street signs) (Lamoureux et al., 2006). Further Rasch analysis suggested the removal of ‘worry about your eyesight getting worse’ due to misfit (Pesudovs et al., 2008) | People with visual impairment including ARMD, retinopathies, glaucoma, other conditions and cataract (Keeffe et al., 1999, Weih et al., 2002, Lamoureux et al., 2006, Lamoureux et al., 2007a, Lamoureux et al., 2007b, Lamoureux et al., 2008b)  Patients awaiting cataract surgery (Pesudovs et al., 2008) | Emotional reaction to vision loss  Household care  Personal care  Leisure and work  Mobility  Social and consumer interactions  Emotional well being  Reading and accessing information  Mobility and independence  (Lamoureux et al., 2007c) | Ordinal 6 point scale – 0 = Not at all to 5 = all the time (14 items)  Ordinal 7 point scale – 0 = Not at all to 5 = Can’t do this because of eyesight plus 8 = Don’t do this for other reasons (19 items). (Hassell et al., 2000)  Rasch 4 point scale – 0 = low participation to 3 = high participation (26 items)  Rasch 3 point scale – 0 = low participation to 2 = high participation (2 items – reading ordinary sized print and getting needed information) (Lamoureux et al., 2006) | Interview (Keeffe et al., 1999, Weih et al., 2002, Lamoureux et al., 2007a, Lamoureux et al., 2007b)  Combination of interview and self-administration (Lamoureux et al., 2006, Lamoureux et al., 2007c, Pesudovs et al., 2008) |
| Indian visual function questionnaire  (IND-VFQ) | To develop a patient defined vision function questionnaire in a population of visually impaired and blind people living in a low income country | Patients with vision loss in a low income country | *Literature review/other instruments:* No  *Lay focus group:* 10 patients per group, 40 specific to diagnosis (cataracts, glaucoma, ARMD, diabetic retinopathy) and 6 mixed groups.  *Expert focus group:* No  *Expert opinion:* No (Murthy et al., 2005) | No | The initial pilot questionnaire (103 items) was tested with the aim of item reduction. Pre-designed exclusion criteria for items – not relevant >10% of patients (18 items excluded) and no difficulty reported by >30% of patients (40 items excluded). Resulting in a 45 item questionnaire. Further item reduction using missing data, redundancy and item convergent/discriminant validity producing the 33 item questionnaire (Gupta et al., 2005).  Rasch analysis resulted in a further 5 item reduction (Finger et al., 2011).  Another Rasch analysis excluded one item from the visual functioning scale or activity limitation subscale (Gothwal et al., 2012a) | Patients with cataract, glaucoma, diabetic retinopathy, ARMD and no visual condition (Gupta et al., 2005) | Visual symptoms  Psychosocial impact  General functioning (Gupta et al., 2005)  Rasch analysis altered subscales:  Mobility  Activity limitation or visual functioning  Visual symptoms  Psychosocial impact (Finger et al., 2011, Gothwal et al., 2012a) | Ordinal 5 point scale – 1 = ‘not at all’ to 4 = ‘a lot’ plus x = ‘unable to carry out because of vision impairment’ (Gupta et al., 2005)  Rasch scale 4 point scale – 1 = ‘not at all’ to 3 = ‘a lot’ plus x = ‘unable to carry out because of vision impairment’ (Finger et al., 2011).  Rasch analysis approved original rating scale (Gothwal et al., 2012a) | Interview (Gupta et al., 2005, Gothwal et al., 2012a) |
| Low vision quality of life questionnaire (LVQoL) | To assess the quality of life in patient with low vision and allow evaluation of rehabilitation strategies | Patients with non-treatable vision loss | *Literature review/other instruments:* Yes – existing questionnaires assessed  *Lay focus group:* No but opinion of patients with low vision sort with MDT.  *Expert focus group:* No  *Expert opinion:* MDT (ophthalmologists, optometrists, orthoptists, occupational therapists, welfare officiers, audiologists and patients with low vision).  (Wolffsohn and Cochrane, 2000) | No | A pilot questionnaire was developed using questions from existing questionnaires found in literature review resulting in 74 items. Pre-designed exclusion criteria for items – not relevant >33% of patients, little or great difficulty by >65% of patients and reliability of items <0.60 – resulted in a reduction to 25 items (Wolffsohn and Cochrane, 2000) | Patients with low vision diagnosed with a variety of conditions | General vision  Mobility  Lighting issues  Psychological adjustment  Reading and fine work  Activities of daily living | Ordinal 6-point scale – 5 = ‘no difficulty’ to 0 = ‘could no longer performed because of vision’ plus a ‘no longer perform because of vision’ and a ‘not relevant’ option (Wolffsohn and Cochrane, 2000) | RCT comparison of postal, telephone or interview (Wolffsohn et al., 2000).  Self-administration (de Boer et al., 2006) |
| Melbourne low vision ADL index (MLVAI) | Assess ability to perform activities of daily living | Patients with low vision | *Literature review/other instruments:* Yes  *Lay focus group:* No  *Expert focus group:* No  *Expert opinion:* No | Unclear | A pilot tool was developed which consisted of nine self-reported items and 18 observed items. Factor analysis resulted in two items (shirt buttoning, naming colours) being eliminated. Rasch analysis was also performed which highlighted redundant items, however, further item reduction did not occur at this stage due to a small sample size (Haymes et al., 2001a).  A weighted version of the tool was created to produce personal impact estimates by completing a personal importance scale(Haymes et al., 2001c) | Adults with ocular disease and stable visual impairment (Haymes et al., 2001a).  Patients with ARMD (Haymes et al., 2001b). |  | Ordinal 5-point scale – 0 = ‘very unsatisfactory’ to 4 = ‘very satisfactory’ (Haymes et al., 2001a) | Clinical assessment (Haymes et al., 2001b, Haymes et al., 2001c) |
| Mobility questionnaire | To assess subjective reporting of mobility function in patient with retinitis pigmentosa | Patients with retinitis pigmentosa | *Literature review/other instruments:* Critical review – no details published  *Lay focus group:* No  *Expert focus group:* No  *Expert opinion:* One experience mobility instructor | No | Forty seven items were chosen and split into two parts. Validity demonstrated by Rasch analysis, no resulting item reduction (Turano et al., 1999) | Patients with retinitis pigmentosa (Turano et al., 1999) and open-angle glaucoma (Turano et al., 2002) | Mobility  Mobility related behaviour | 5-point scale – 1 = ‘no difficulty’ to 5 = ‘extreme difficulty’ plus ‘not applicable’ (Turano et al., 1999) | Self-administered (Turano et al., 1999)  Interview (Turano et al., 2002) |
| National Eye Institute Refractive Error Correction quality of life Questionnaire  (NEI RQL) | To assess vision-targeted health-related quality of life for persons with well corrected refractive error | Patients with refractive error | *Literature review/other instruments:* Yes  *Lay focus group:* Yes, 52 groups with a mean of 8 patients per group  *Expert focus group:* No  *Expert opinion:* No | No | A pilot questionnaire (94 items) created using focus groups were reduced during testing to a 42-item questionnaire (Hays et al., 2003). Rasch analysis highlighted 13 misfitting items and suggested the removal of these items (McAlinden et al., 2011a) | Patients requiring refractive correction and emmetropes (Hays et al., 2003).  Patients awaiting refractive surgery (McAlinden et al., 2011a) | Clarity of vision  Expectations  Near vision  Far vision  Diurnal fluctuations  Activity limitations  Glare  Symptoms  Dependence on corrections  Worry  Suboptimal correction  Appearance  (Hays et al., 2003) | Sixteen options of response scale depending on item, varying between dichotomous and 6-point ordinal scale (Hays et al., 2003)  Rasch analysis highlighted the need to remove categories 2 and 3 in the 6-point scales to create a 4-point scale (McAlinden et al., 2011a) | Self-administered (Hays et al., 2003, McAlinden et al., 2011a) |
| National Eye Institute Visual Functioning Questionnaire  (NEI VFQ)  and  Long form visual functioning scale (LFVFS-39) | To assess the impact of visual impairment on health related quality of life across various eye conditions | Patients with visual impairment | *Literature review/other instruments:* Existing questionnaires  *Lay focus group:* 246 eye clinic patients with various ophthalmic diagnoses  *Expert focus group:* No  *Expert opinion:* No | NEI-VFQ: Unclear  Neuro-10: Yes | Focus group analysis resulted in the 51-item pilot version of the NEI-VFQ (Mangione et al., 1998a).  The pilot version underwent item reduction by qualitative criteria and linear regression to resulting in 25-items (Mangione et al., 1998b).  A 10-item Neuro-Ophthalmic Supplement (Neuro-10) to the NEI-VFQ 25 was developed using survey and focus group methods. A decision to delay item reduction (possibly – ‘eye/eyelid appearance is unusual’ and ‘ptosis’) until further testing has taken place (Ma et al., 2002, Raphael et al., 2006).  Ceiling effects were found in three subscales: general vision, ocular pain and vision-specific mental health.  Rasch analysis was performed on a 7-item version of the questionnaire created by author’s choice (Ryan et al., 2008).  The NEI VFQ was re-engineered using Rasch analysis and renamed Long-Form Visual Functioning Scale (LFVFS) with the number items being reduced to 8 and Short-Form Visual Functioning Scale (SFVFS) with 6 items (Pesudovs et al., 2010a).  Rasch analysis of the 25 item version suggested item reduction resulting in a 19-item version (Marella et al., 2010). Rasch analysis has also been used to create a 6-item utility index (Kowalski et al., 2012) | Multi-condition population (cataracts, ARMD, diabetic retinopathy, glaucoma and low vision) (Mangione et al., 1998b, Mangione et al., 2001, Clemons et al., 2003).  Optic neuritis (Cole et al., 2000).  Multi-condition and visually normal population (Globe et al., 2003).  Multiple sclerosis and other neuro-ophthalmic disorders with Neuro-10 supplement (Raphael et al., 2006).  Patients with cataracts (Pesudovs et al., 2010a).  Age-related macular degeneration (Orr et al., 2011) . Uveitis (Naik et al., 2013) | General health  General vision  Near visual activities  Distance visual activities  Ocular pain  Vision specific social function  Vision specific role difficulties  Vision specific mental health  Vision specific dependency  Driving  Colour vision  Peripheral vision  Reduced to four subscales using Rasch analysis:  General health  Near activities  Distance activities  Role difficulties  (Pesudovs et al., 2010a)  An alternative subscale structure was suggested:  Visual functioning  Socioemotional  (Marella et al., 2010) | Ordinal 5-point scale – 1 = ‘all of the time’ to 5 = ‘none of the time’ or 1 = ‘no difficulty at all’ to 5 = ‘stopped doing because of your eyesight’ plus a ‘stopped doing for other reasons or not interested in doing this option’  Rasch analysis recommended the collapse of categories 1 and 2 to create a 4-point scale (Massof, 2007)  Rasch analysis suggested collapsing options: no difficulty = 0 and a little difficulty = 1 (Ryan et al., 2008)  Rasch analysis suggested collapsing middle options to create a dichotomous scale: 1 = always and 2 = never , 1 = true and 2 = false plus the not sure option (Marella et al., 2010) | Interview (Mangione et al., 1998b, Mangione et al., 2001, Globe et al., 2003, Marella et al., 2010, Lloyd et al., 2013)  Self-administered  (Cole et al., 2000, Raphael et al., 2006, Ryan et al., 2008, Pesudovs et al., 2010a) |
| Nursing home vision targeted health related quality of life questionnaire (NHVQoL) | Evaluate vision-targeted health-related quality of life in older adults who live in nursing homes | Older adults who live in nursing homes | *Literature review/other instruments:* Yes  *Lay focus group:* No Structured interviews  *Expert focus group:* No  *Expert opinion:* No | Unclear | A pilot questionnaire with 57 items was developed using themes which emerged from interviews (Dreer et al., 2007).  An adapted version of the questionnaire was created to produce personal impact by the addition of bother subscales as a part B to the original version (Elliott et al., 2010) | Residents of nursing homes (Dreer et al., 2007, Lamoureux et al., 2009a) | Reading  Ocular symptoms  General vision  ADLs  Mobility  Social activities/hobbies  Psychological distress  Adaptation/coping  Social interaction  During Rasch analysis the adaptation/coping subscale was added to psychological distress (Lamoureux et al., 2009a) | 5-point scale – ‘no difficulty at all’ to ‘stopped doing this because of your eyesight’ or ‘none of the time’ to ‘all of the time’ or ‘definitely true’ to ‘definitely false’ plus many items had additional options of ‘not sure’, ‘stopped doing this for other reasons or not interested in doing this’ and ‘could do this but not given the opportunity’ (Dreer et al., 2007) | Interview (Dreer et al., 2007, Lamoureux et al., 2009a, Elliott et al., 2010) |
| QoL and visual function questionnaire  (QoL-VFQ) | To evaluate self-reported visual satisfaction | Patients with chronic eye disease causing visual impairment | *Literature review/other instruments:* Yes  *Lay focus group:* No  *Expert focus group:* No  *Expert opinion:* No | No | A pilot questionnaire was developed consisting of 17 questions selected by consensus of the authors (Carta et al., 1998). Rasch analysis required the reduction of two items (lights of oncoming cars and recognising colour) resulting in a 15-item questionnaire (Gothwal et al., 2009c) | Patients with cataract, glaucoma, ARMD, branch retinal vein occlusion, minor refractive error or presbyopia (Carta et al., 1998)  Patients awaiting cataract surgery (Gothwal et al., 2009c) | Overall self-assessment of visual satisfaction  Self-assessment of visual field  Self-assessment of distance visual acuity  Self-assessment of near visual acuity  Self-assessment of sensory adaptation  Self-assessment of colour vision  Rasch analysis revealed poor performance of the subscales and recommended addition of items to subscales or that the subscales not be used (Gothwal et al., 2009c) | 3-point scale – 1 = ‘not at all’ to 3 = ‘very much’ (Carta et al., 1998)  Rasch analysis confirmed optimal use of the scale (Gothwal et al., 2009c) | Interview (Carta et al., 1998)  Self-administration (Gothwal et al., 2009c) |
| Quality of vision (QoV) | Evaluate quality of vision | Patients with or without refractive correction and/or eye disease | *Literature review/other instruments:* Yes  *Lay focus group:* Yes x3 (5 non-experts) plus 15 subject interviews  *Expert focus group:* Yes x3 (5 experts)  *Expert opinion:* No | No | A 23 items underwent discussion within a focus group for item redundancy, representation and face validity to reduce items to create a 30-item questionnaire focused on 10 symptoms. The pilot questionnaire developed was tested using conventional statistics and Rasch analysis (McAlinden et al., 2010) | Patients with or without refractive correction and patients with cataracts (McAlinden et al., 2010).  Patients awaiting refractive surgery (McAlinden et al., 2011b) | Frequency  Severity  Bothersomeness  Further analysis of the subscales recommended continuing using all three (McAlinden et al., 2013) | 4-point scale  Frequency 0 = ‘Never’ to 3 = ‘Very often’  Severity 0 = ‘Not at all’ to 3 = ‘ Severe’  Bothersomeness 0 = ‘Not at all’ to 3 = ‘Very’  (McAlinden et al., 2010) | Self-administration (Piermarocchi et al., 2011) |
| Self-report assessment of functional visual performance  (SRA-VFA) | Measure the performance of vision dependant ADLs in older adults with low vision | Older adults with age related ocular disease | *Literature review/other instruments:* No  *Lay focus group:* No  *Expert focus group:* No  *Expert opinion:* Yes | No | A group of experts developed a list of common ADLs and a five-point rating scale. A separate panel of experts reviewed the list and rating scale. The pilot questionnaire consisted of 39 items (Gilbert and Baker, 2011) | Patients with homonymous hemianopia (Mennem et al., 2012). | Reading  Writing  Money management  Telephone usage  Reading a timepiece  Personal care  Clothing care  Meal Preparation  Leisure  Functional mobility  Reading  Writing  Communication  Financial and health management  Feeding  Personal hygiene  Dressing  Clothing care  Meal Preparation  Shopping  Functional mobility  Community or social and leisure participation | 5-point scale – 1 = ‘unable’ to 5 = ‘independent’ plus a ‘not applicable’ option  Rasch analysis suggested the need to collapse the middle ratings (2, 3 and 4) to create: a 3-point scale – 1 = ‘unable: dependant on other to perform task, would perform task if able’ to 3 = ‘independent: experiences no difficulty performing task safely, accurately and efficiently’ (Velozo et al., 2013) | Interview (Mennem et al., 2012, Velozo et al., 2013) |
| Severity of visual field damage | To assess subjective disability associated with visual field loss | Patients with glaucoma | *Literature review/other instruments:* No  *Lay focus group:* Yes - interviews  *Expert focus group:* No  *Expert opinion:* Yes – physicians and visual field technicians | No | The original question consisted of 15 items developed from expert and patient input (Mills and Drance, 1986). The questionnaire was modified, rewording of questions and the removal of items resulted in a 10-item questionnaire (Viswanathan et al., 1999) | Patients with glaucoma (Mills and Drance, 1986, Viswanathan et al., 1999) | - | Ordinal 3-point scale 1 = no, 2 = uncertain and 3 = yes (Mills and Drance, 1986) | Interview (Mills and Drance, 1986, Viswanathan et al., 1999) |
| Veterans affairs low vision visual functioning questionnaire  (VA LV VFQ) | To measure functional ability of low vision patients and measure patient-centred outcomes of low vision rehabilitation | Veterans with low vision | *Literature review/other instruments:* Yes – clinical guidelines  *Lay focus group:* No – structure interviews with patients  *Expert focus group:* No  *Expert opinion:* Yes – consensus panel recommendations | No | The second round of modified Delphi analysis selected 48 items for the pilot questionnaire (Stelmack et al., 2004a). The questionnaire was tested using Rasch analysis (Szlyk et al., 2004). Some items were identified as poor fit during Rasch analysis, however, any item change was deferred (Stelmack et al., 2004b). A short form of the questionnaire was developed by reducing items based on Rasch analysis and clinical relevance, resulting in a 20 item questionnaire (Stelmack et al., 2006, Stelmack and Massof, 2007) | Patients with low vision (ARMD, glaucoma, diabetic retinopathy and other) (Stelmack et al., 2004a, Stelmack et al., 2004b, Szlyk et al., 2004).  Patient with macular disease (Stelmack and Massof, 2007).  Patients with homonymous hemianopia (George et al., 2011) | Visual ability  Mobility  Reading  Visual motor  Visual information | Identical response scales for all items: 5-point scale – 1 = ‘not difficult’ to 5 = ‘impossible’  Rasch analysis indicated the collapse of categories 2 ‘slightly difficult’ and 3 ‘moderately difficult’ resulting in a 4-point scale (Stelmack et al., 2004b) | Interview (Stelmack et al., 2004a, Stelmack et al., 2004b, Szlyk et al., 2004) |
| Vision and quality of life index (VisQoL) | Economic evaluation of eye care and rehabilitation programs | Patients with visual impairment | *Literature review/other instruments:* Yes  *Lay focus group:* Yes – 3 groups with 8-9 patients  *Expert focus group:* No  *Expert opinion:* No | No | An item bank of 33 was created from the results of the focus groups. Item reduction was achieved through factor analysis and item response theory. A pilot study was then conducted using this item bank. Factor analysis retained 13 items. The final version contained 6 items following iterative structural equation modelling (Misajon et al., 2005) | Patients with visual impairment (Misajon et al., 2005) | Physical well-being  Independence  Social well-being  Emotional well-being  Self-actualisation  Planning and organisation | Range of between 5 and 7-point scales, different for each item (Misajon et al., 2005) | Self-administration (Misajon et al., 2005) |
| Vision function and quality of life questionnaires  (VF and QOL) | To assess improvement in functioning following cataract surgery | Patients with cataracts | *Literature review/other instruments:* Yes  *Lay focus group:* No  *Expert focus group:* No  *Expert opinion:* Yes – 3 ophthalmologists and 2 social workers | No | Two pilot questionnaires VF (13 items) and QOL (12 items) were developed and tested (Fletcher et al., 1997) (Fletcher et al., 1997).  Rasch analysis found the VF questionnaire to be valid. However, despite removal of items the QOL questionnaire was found to be limiting precision and could not be deemed valid (Gothwal et al., 2009d) | Patients with cataract, glaucoma, iritis, ARMD or corneal disease (Fletcher et al., 1997).  Patients with cataracts (Gothwal et al., 2009d) | Overall visual function  Visual perception  Limitation in everyday activities and visual acuity  Peripheral vision  Sensory adaptation, light dark adaptation, visual search, colour discrimination and glare disability  Depth perception  Self-care  Mobility  Social  Mental  (Fletcher et al., 1997).  Rasch analysis recommended these subscales not to be used (Gothwal et al., 2009d) | Ordinal 4 point scale – 1 = ‘not at all’ to = ‘a lot’ (Fletcher et al., 1997). | Interview (Fletcher et al., 1997)  Self-administration (Gothwal et al., 2009d) |
| Vision related quality of life (VQoL)  or  Vision-related quality of life core measure  (VCM1) | To assess vision related quality of life | Patients with visual impairment | *Literature review/other instruments:* Yes  *Lay focus group:* No – semi structured interviews with 38 patients  *Expert focus group:* No - 11 interviews with support workers and professionals  *Expert opinion:* Consultation with 26 professionals | No | Pre-testing finalised the ‘parent’ version with of the questionnaire containing 139 items. The selection of items took a modular approach to enable to questionnaire to meet the requirements of different groups of patients. Ten items were identified as core items and named VCM1(Frost et al., 1998). Rasch analysis did not change any included items of the VCM1 (Lamoureux et al., 2008c) | Patients with cataract, ARMD, Glaucoma, Aphakia, Amblyopia, Corneal lesions, Diabetic retinopathy, Thyroid eye disease, Retinal detachment, Iritis, Ocular hypertension, Ocular trauma, ocular tumour, Optic neuropathy, RP, Retinal vascular occlusions and no pathology (Frost et al., 1998).  Patients awaiting cataract surgery  (Lamoureux et al., 2008c) | Overall vision  Visual symptoms  Self esteem  Emotion  Safety  General  Self-care  Domestic  Financial  Reading  Miscellaneous information  Mobility  Social interaction  Leisure | Ordinal 6 point scale – 0 = ‘not at all’ to 5 = ‘can’t do because of eye-sight’ plus a ‘don’t do for other reasons’ option  Ordinal 5 point scale – 0 = ‘not at all’ to 5 = ‘all the time’ – the latter used by all VCM1 items (Frost et al., 1998)  Rasch analysis indicated the need to collapse categories 4 ‘very rarely’ and 5 ‘all the time’ resulting in a 4-point scale (Lamoureux et al., 2008c) | Self-administered (de Boer et al., 2006) |
| Visual activity questionnaire (VAQ) | To assess individual’s problems in performing visual activities typical of everyday life | Elderly population | *Literature review/other instruments:* No  *Lay focus group:* No  *Expert focus group:* No  *Expert opinion:* No | No | The pilot questionnaire contained 100 items spilt into 10 areas. Items were reduced by removing those that did not provide further information and items with unclear wording. Factor analysis resulted in reduction of items to 33 (Sloane et al., 1992).  Rasch analysis reduced the number of items to 13 due to multidimensionality. This reduction in items results in floor-effects (Gothwal et al., 2009e) | Adults aged 17 to 89.  Drivers aged 55 and over. (Sloane et al., 1992)  Patients with cataract (Gothwal et al., 2009e) | Glare disability  Light and dark adaptation  Acuity and spatial vision  Visual search  Visual processing speed  Depth perception  Colour discrimination  Peripheral vision  Reduced to in the 13-item version:  Visual search  Visual processing speed  Depth perception  Peripheral vision  (Gothwal et al., 2009e) | Ordinal 5 point scale – 1 = ‘never’ to 5 = ‘always’ (Sloane et al., 1992)  Rasch analysis recommended the collapse of categories 2 and 3 resulting in a 4-point scale (Massof, 2007) | Self-administration (Gothwal et al., 2009e) |
| Visual disability assessment (VDA) | Assess subjective visual disability | Patients with cataracts | *Literature review/other instruments:* Yes  *Lay focus group:* No  *Expert focus group:* No  *Expert opinion:* No | No | A pilot questionnaire of 37 items was developed. Item reduction was performed by eliminating redundant items, resulting in an 18 item questionnaire (Pesudovs and Coster, 1998) | Patients with cataracts (Pesudovs and Coster, 1998, Pesudovs et al., 2010b) | Mobility  Distance/Lighting/Reading  Near and related tasks  (Pesudovs and Coster, 1998)  In order to achieve unidimensionality Rasch analysis suggested the segregation of the mobility subscale and activity limitation subscale (Pesudovs et al., 2010b) | 4 point scale – 1 =’Not at all’ to 4 = ‘A lot’ (Pesudovs and Coster, 1998) | Self-administration (Pesudovs et al., 2010b) |
| Visual disability questionnaire (VDQ) | Aid with prioritising rehabilitation goals | Patients with visual impairment | *Literature review/other instruments:* Yes  *Lay focus group:* Yes – 3 groups (8 participants per group)  *Expert focus group:* No  *Expert opinion:* Yes | No | A pilot questionnaire (28 items) was created. The pilot testing resulted in removal unnecessary items and addition of new items (Marella et al., 2009) | Patients with retinitis pigmentosa, macular degeneration, diabetic retinopathy, glaucoma, optic atrophy, refractive error, corneal disorders, developmental disorders, albinism and other diseases | - | Ordinal 5 point scale – 0 = ‘not important’ to 4 = ‘extremely important’ and ordinal 5 point scale – 0 = ‘not difficult’ to 4 = ‘impossible’  Rasch analysis suggested the collapse of categories 1 and 2 resulting in a 4-point scale (Marella et al., 2009) | Interview (Marella et al., 2009) |
| Visual function index (VFI) | Evaluate the amount of improvement obtained by cataract surgery | Patients awaiting cataract surgery | *Literature review/other instruments:* No  *Lay focus group:* No  *Expert focus group:* No  *Expert opinion:* Yes | No | Items were chosen from those used to routinely interview cataract patients but with the aim of standardisation an 11-item questionnaire was created (Bernth-Petersen, 1985). Rasch analysis found this questionnaire not valid for use in a modern cataract population in a developed country (Gothwal et al., 2010a) | Patients awaiting cataracts surgery (Bernth-Petersen, 1985, Gothwal et al., 2010a) | Direct visual limitations  Mobility limitations  Social role limitations (Bernth-Petersen, 1985) | Dichotomous – ‘yes/no’ or ‘sufficient/insufficient’  Only two items use an ordinal 3-point scale. (Bernth-Petersen, 1985) | Self-administration (Gothwal et al., 2010a) |
| Visual functioning 14 items (VF-14) | To measure functional impairment caused by cataract | Patients with cataracts awaiting surgery | *Literature review/other instruments:* Existing vision related questionnaires  *Lay focus group:* No – individual interviews with patients were conducted  *Expert focus group:* 11 members of National Advisory Panel of ophthalmologists and optometrists  *Expert opinion:* Three ophthalmologists | No | A pilot instrument was developed following the expert focus group. Factor analysis divided items into 4 factors (Steinberg et al., 1994). Various shortened versions have been proposed (Uusitalo et al., 1999, Friedman et al., 2002, Pager, 2004, Moghimi et al., 2007). A reduction in items (large print, fine handwork, and taking part in sports) created the VF-11, this was examined using Rasch analysis (Lamoureux et al., 2008a). Further Rasch analysis reduced items (driving at night and driving in daytime) to the VF-9 with a change of scale (Lamoureux et al., 2009b). Additional revision (eliminating large print, recognising people, sports, cooking and driving) created the VF-8R using a Rasch scale (Gothwal et al., 2010c). | Patients awaiting cataract surgery (Steinberg et al., 1994)  Patients with retinal disease (Linder et al., 1999)    Population based study (Lamoureux et al., 2008a) | Seeing steps  Writing cheques  Playing table games  Taking part in sports  Cooking  Reading small print  Doing fine handwork  Reading a newspaper  Daytime driving  Night-time driving  Reading street signs  Reading large print  Recognising people  Watching television | Ordinal 5 point scale – 0 =’Unable to perform activity’ to 4 = ‘No difficulty’ (Steinberg et al., 1994, Massof, 2007)  Reduced to a 4-point scale  Combining points 2 and 3 (Lamoureux et al., 2009b) | Interview (Cassard et al., 1995, Lamoureux et al., 2008a, Lamoureux et al., 2009b)  Self-administration (Gothwal et al., 2010c) |
| Visual symptom and quality of life questionnaire (VSQ) | To assess the outcome following second-eye cataract surgery | Patients awaiting second-eye cataract surgery | *Literature review/other instruments:* Yes  *Lay focus group:* No – individual interviews with 40 patients were conducted  *Expert focus group:* No  *Expert opinion:* Yes – 6 ophthalmologists, 6 ophthalmic nurses, 4 optometrists, 1 social worker and 1 OT | No | A 32-item draft questionnaire was devised using the data obtained from clinical experts and patients. Results from pilot testing resulted in some reorganisation and rewording of items, most commonly clarifying the question with ‘in the past month’ and ‘because of trouble with your eyesight’. Factor analysis resulted in the removal of four items (perception of colour, diplopia, blinkered vision and watering eyes). A short (14-item) and a long (26-item) final version were created (Donovan et al., 2003). Rasch analysis was performed on the 26-item version, due to lack of unidimensionality all items from the subscales visual symptoms and vision-specific quality of life were removed. Two further items were removed from the remaining subscale due to misfit, creating a 14-item questionnaire (Gothwal et al., 2011a) | Patients awaiting cataract surgery or recently undergone cataract surgery (Donovan et al., 2003, Gothwal et al., 2011a) | Visual symptoms/disability  Vision-specific quality of life | Ordinal 4 or 5 point scale – 13 formats (Donovan et al., 2003)  Rasch analysis indicated the collapse of options 3 and 4 for the question ‘do you have difficulty recognising people’s faces’ (Gothwal et al., 2011a) | Self-administration (Gothwal et al., 2011a) |

AHMADIAN, L. & MASSOF, R. 2008. Impact of general health status on validity of visual impairment measurement. *Ophthalmic Epidemiology,* 15**,** 345-55.

BERNTH-PETERSEN, P. 1985. Visual functioning in cataract patients. Methods of measuring and results. *Acta Ophthalmologica,* 59**,** 198-205.

CARTA, A., BRACCIO, L., BELPOLITI, L., SOLIANI, L., SARTORE, F., GANDOLFI, S. A. & MARAINI, G. 1998. Self-assessment of the quality of vision: association of questionnaire score with objective clinical tests. *Current Eye Research,* 17**,** 506-512.

CASSARD, S. D., PATRICK, D. L., DAMIANO, A. M. & ET AL. 1995. Reproducibility and responsiveness of the vf-14: An index of functional impairment in patients with cataracts. *Archives of Ophthalmology,* 113**,** 1508-1513.

CLEMONS, T. E., CHEW, E. Y., BRESSLER, S. B., MCBEE, W. & AGE-RELATED EYE DISEASE STUDY RESEARCH, G. 2003. National Eye Institute Visual Function Questionnaire in the Age-Related Eye Disease Study (AREDS): AREDS Report No. 10. *Archives of Ophthalmology,* 121**,** 211-7.

COLE, S. R., BECK, R. W., MOKE, P. S., GAL, R. L. & LONG, D. T. 2000. The National Eye Institute Visual Function Questionnaire: experience of the ONTT. Optic Neuritis Treatment Trial. *Investigative Ophthalmology & Visual Science,* 41**,** 1017-21.

DE BOER, M. R., TERWEE, C. B., DE VET, H. C., MOLL, A. C., VOLKER-DIEBEN, H. J. M. & VAN RENS, G. H. M. B. 2006. Evaluation of cross-sectional and longitudinal construct validity of two vision-related quality of life questionnaires: The LVQOL and VCM1. *Quality of Life Research,* 15**,** 233-248.

DENNY, F., MARSHALL, A. H., STEVENSON, M. R., HART, P. M. & CHAKRAVARTHY, U. 2007. Rasch analysis of the daily living tasks dependent on vision (DLTV). *Investigative Ophthalmology & Visual Science,* 48**,** 1976-82.

DONOVAN, J. L., BROOKES, S. T., LAIDLAW, D. A., HOPPER, C. D., SPARROW, J. M. & PETERS, T. J. 2003. The development and validation of a questionnaire to assess visual symptoms/dysfunction and impact on quality of life in cataract patients: the Visual Symptoms and Quality of life (VSQ) Questionnaire. *Ophthalmic Epidemiology,* 10**,** 49-65.

DREER, L. E., MCGWIN, G., JR., SCILLEY, K., MEEK, G. C., DYER, A., SEKER, D. & OWSLEY, C. 2007. Development of a nursing home vision-targeted health-related quality of life questionnaire for older adults. *Aging & Mental Health,* 11**,** 722-33.

ELLIOTT, A. F., DREER, L. E., MCGWIN, G., JR., SCILLEY, K. & OWSLEY, C. 2010. The personal burden of decreased vision-targeted health-related quality of life in nursing home residents. *Journal of Aging & Health,* 22**,** 504-521.

FELIUS, J., BEAUCHAMP, G. R., STAGER, D. R., SR., VAN DE GRAAF, E. S. & SIMONSZ, H. J. 2007. The Amblyopia and Strabismus Questionnaire: English translation, validation, and subscales. *American Journal of Ophthalmology,* 143**,** 305-310.

FINGER, R. P., KUPITZ, D. G., HOLZ, F. G., BALASUBRAMANIAM, B., RAMANI, R. V., LAMOUREUX, E. L. & FENWICK, E. 2011. The impact of the severity of vision loss on vision-related quality of life in India: an evaluation of the IND-VFQ-33. *Investigative Ophthalmology & Visual Science,* 52**,** 6081-8.

FLETCHER, A. E., ELLWEIN, L. B., SELVARAJ, S., VIJAYKUMAR, V., RAHMATHULLAH, R. & THULASIRAJ, R. D. 1997. Measurements of vision function and quality of life in patients with cataracts in southern India. Report of instrument development. *Archives of Ophthalmology,* 115**,** 767-74.

FRIEDMAN, D. S., TIELSCH, J. M., VITALE, S., BASS, E. B., SCHEIN, O. D. & STEINBERG, E. P. 2002. VF-14 item specific responses in patients undergoing first eye cataract surgery: can the length of the VF-14 be reduced? *British Journal of Ophthalmology,* 86**,** 885-891.

FROST, N. A., SPARROW, J. M., DURANT, J. S., DONOVAN, J. L., PETERS, T. J. & BROOKES, S. T. 1998. Development of a questionnaire for measurement of vision-related quality of life. *Ophthalmic Epidemiology,* 5**,** 185-210.

GEORGE, S., HAYES, A., CHEN, C. & CROTTY, M. 2011. Are vision-specific quality of life questionnaires important in assessing rehabilitation for patients with hemianopia post stroke? *Topics in Stroke Rehabilitation,* 18**,** 394-401.

GILBERT, M. P. & BAKER, S. S. 2011. Evaluation and intervention for basic and instrumental activities of daily living. *In:* WARREN, M. (ed.) *Occupational therapy interventions for adults with low vision.* Bethesda, Maryland: AOTA Press.

GLOBE, D., VARMA, R., AZEN, S. P., PAZ, S., YU, E., PRESTON-MARTIN, S. & LOS ANGELES LATINO EYE STUDY, G. 2003. Psychometric performance of the NEI VFQ-25 in visually normal Latinos: the Los Angeles Latino Eye Study. *Investigative Ophthalmology & Visual Science,* 44**,** 1470-8.

GOTHWAL, V. K., BAGGA, D. K. & SUMALINI, R. 2012a. Rasch analysis of the Indian vision function questionnaire. *British Journal of Ophthalmology,* 96**,** 619-23.

GOTHWAL, V. K., REDDY, S. P., BHARANI, S., BAGGA, D. K., SUMALINI, R., GARUDADRI, C. S., RAO, H. L., SENTHIL, S., PATHAK-RAY, V. & MANDAL, A. K. 2012b. Impact of glaucoma on visual functioning in Indians. *Investigative Ophthalmology & Visual Science,* 53**,** 6081-92.

GOTHWAL, V. K., WRIGHT, B. D., LAMOUREUX, E. L. & PESUDOVS, K. 2009a. Validity of the adaptation to age-related vision loss scale in an Australian cataract population. *Journal of Optometry,* 2**,** 142-147.

GOTHWAL, V. K., WRIGHT, T., LAMOUREUX, E. L. & PESUDOVS, K. 2010a. Psychometric properties of visual functioning index using Rasch analysis. *Acta Opthalmologica,* 88**,** 797-803.

GOTHWAL, V. K., WRIGHT, T. A., LAMOUREUX, E. L., KHADKA, J., MCALINDEN, C. & PESUDOVS, K. 2011a. Improvements in visual ability with first-eye, second-eye, and bilateral cataract surgery measured with the visual symptoms and quality of life questionnaire. *Journal of Cataract & Refractive Surgery,* 37**,** 1208-16.

GOTHWAL, V. K., WRIGHT, T. A., LAMOUREUX, E. L., LUNDSTROM, M. & PESUDOVS, K. 2009b. Catquest questionnaire: re-validation in an Australian cataract population. *Clinical & Experimental Ophthalmology,* 37**,** 785-94.

GOTHWAL, V. K., WRIGHT, T. A., LAMOUREUX, E. L. & PESUDOVS, K. 2009c. Rasch analysis of the quality of life and vision function questionnaire. *Optometry & Vision Science,* 86**,** E836-44.

GOTHWAL, V. K., WRIGHT, T. A., LAMOUREUX, E. L. & PESUDOVS, K. 2009d. Rasch analysis of visual function and quality of life questionnaires. *Optometry & Vision Science,* 86**,** 1160-8.

GOTHWAL, V. K., WRIGHT, T. A., LAMOUREUX, E. L. & PESUDOVS, K. 2009e. Visual Activities Questionnaire: assessment of subscale validity for cataract surgery outcomes. *Journal of Cataract & Refractive Surgery,* 35**,** 1961-9.

GOTHWAL, V. K., WRIGHT, T. A., LAMOUREUX, E. L. & PESUDOVS, K. 2010b. Activities of Daily Vision Scale: what do the subscales measure? *Investigative Ophthalmology & Visual Science,* 51**,** 694-700.

GOTHWAL, V. K., WRIGHT, T. A., LAMOUREUX, E. L. & PESUDOVS, K. 2010c. Measuring outcomes of cataract surgery using the Visual Function Index-14. *Journal of Cataract & Refractive Surgery,* 36**,** 1181-8.

GOTHWAL, V. K., WRIGHT, T. A., LAMOUREUX, E. L. & PESUDOVS, K. 2011b. Multiplicative rating scales do not enable measurement of vision-related quality of life. *Clinical & Experimental Optometry,* 94**,** 52-62.

GUPTA, S. K., VISWANATH, K., THULASIRAJ, R. D., MURTHY, G. V., LAMPING, D. L., SMITH, S. C., DONOGHUE, M. & FLETCHER, A. E. 2005. The development of the Indian vision function questionnaire: field testing and psychometric evaluation. *British Journal of Ophthalmology,* 89**,** 621-7.

HART, P. M., CHAKRAVARTHY, U., STEVENSON, M. R. & JAMISON, J. Q. 1999. A vision specific functional index for use in patients with age related macular degeneration. *British Journal of Ophthalmology,* 83.

HART, P. M., STEVENSON, M. R., MONTGOMERY, A. M., MULDREW, K. A. & CHAKRAVARTHY, U. 2005. Further validation of the Daily Living Tasks Dependent on Vision: identification of domains. *British Journal of Ophthalmology,* 89**,** 1127-30.

HASSELL, J. B., WEIH, L. M. & KEEFFE, J. E. 2000. A measure of handicap for low vision rehabilitation: the impact of vision impairment profile. *Clinical & Experimental Ophthalmology,* 28**,** 156-61.

HATT, S. R., LESKE, D. A., BRADLEY, E. A., COLE, S. R. & HOLMES, J. M. 2009. Development of a quality-of-life questionnaire for adults with strabismus. *Ophthalmology,* 116**,** 139-144.e5.

HAYMES, S. A., JOHNSTON, A. W. & HEYES, A. D. 2001a. The development of the Melbourne low-vision ADL index: a measure of vision disability. *Investigative Ophthalmology & Visual Science,* 42**,** 1215-25.

HAYMES, S. A., JOHNSTON, A. W. & HEYES, A. D. 2001b. Preliminary investigation of the responsiveness of the Melbourne Low Vision ADL index to low-vision rehabilitation. *Optometry & Vision Science,* 78**,** 373-80.

HAYMES, S. A., JOHNSTON, A. W. & HEYES, A. D. 2001c. A weighted version of the Melbourne Low-Vision ADL Index: a measure of disability impact. *Optometry & Vision Science,* 78**,** 565-79.

HAYS, R. D., MANGIONE, C. M., ELLWEIN, L., LINDBLAD, A. S., SPRITZER, K. L. & MCDONNELL, P. J. 2003. Psychometric properties of the National Eye Institute-Refractive Error Quality of Life instrument. *Ophthalmology,* 110**,** 2292-301.

HOLMES, J. M., LESKE, D. A. & KUPERSMITH, M. J. 2005. New methods for quantifying diplopia. *Ophthalmology,* 112**,** 2035-9.

HOLMES, J. M., LIEBERMANN, L., HATT, S. R., SMITH, S. J. & LESKE, D. A. 2013. Quantifying diplopia with a questionnaire. *Ophthalmology,* 120**,** 1492-6.

HOROWITZ, A. & REINHARDT, J. P. 1998. Development of the adaptation to age-realted vision loss scale. *Journal of Visual Impairment and Blindness,* 92**,** 30-41.

HOROWITZ, A., REINHARDT, J. P. & RAYKOV, T. 2007. Development and validation of a short-form adaptation of the age-related vision loss scale: The AVL12. *Journal of Visual Impairment and Blindness,* 101**,** 146-159.

KEEFFE, J. E., LAM, D., CHEUNG, A., DINH, T. & MCCARTY, C. A. 1998. Impact of vision impairment on functioning. *Australian & New Zealand Journal of Ophthalmology,* 26**,** S16-S18.

KEEFFE, J. E., MCCARTY, C. A., HASSELL, J. B. & GILBERT, A. G. 1999. Description and measurement of handicap caused by vision impairment. *Australian & New Zealand Journal of Ophthalmology,* 27**,** 184-6.

KHADKA, J., PESUDOVS, K., MCALINDEN, C., VOGEL, M., KERNT, M. & HIRNEISS, C. 2011. Reengineering the glaucoma quality of life-15 questionnaire with rasch analysis. *Investigative Ophthalmology & Visual Science,* 52**,** 6971-7.

KOWALSKI, J. W., RENTZ, A. M., WALT, J. G., LLOYD, A., LEE, J., YOUNG, T. A., CHEN, W. H., BRESSLER, N. M., LEE, P., BRAZIER, J. E., HAYS, R. D. & REVICKI, D. A. 2012. Rasch analysis in the development of a simplified version of the National Eye Institute Visual-Function Questionnaire-25 for utility estimation. *Quality of Life Research,* 21**,** 323-34.

LAMOUREUX, E. L., CHONG, E. W., THUMBOO, J., WEE, H. L., WANG, J. J., SAW, S. M., AUNG, T. & WONG, T. Y. 2008a. Vision impairment, ocular conditions, and vision-specific function: the Singapore Malay Eye Study. *Ophthalmology,* 115**,** 1973-81.

LAMOUREUX, E. L., FENWICK, E., MOORE, K., KLAIC, M., BORSCHMANN, K. & HILL, K. 2009a. Impact of the severity of distance and near-vision impairment on depression and vision-specific quality of life in older people living in residential care. *Investigative Ophthalmology & Visual Science,* 50**,** 4103-9.

LAMOUREUX, E. L., FERRARO, J. G., PALLANT, J. F., PESUDOVS, K., REES, G. & KEEFFE, J. E. 2007a. Are standard instruments valid for the assessment of quality of life and symptoms in glaucoma? *Optometry & Vision Science,* 84**,** 789-96.

LAMOUREUX, E. L., HOOPER, C. Y., LIM, L., PALLANT, J. F., HUNT, N., KEEFFE, J. E. & GUYMER, R. H. 2007b. Impact of cataract surgery on quality of life in patients with early age-related macular degeneration. *Optometry & Vision Science,* 84**,** 683-8.

LAMOUREUX, E. L., PALLANT, J. F., PESUDOVS, K., HASSELL, J. B. & KEEFFE, J. E. 2006. The Impact of Vision Impairment Questionnaire: an evaluation of its measurement properties using Rasch analysis. *Investigative Ophthalmology & Visual Science,* 47**,** 4732-41.

LAMOUREUX, E. L., PALLANT, J. F., PESUDOVS, K., REES, G., HASSELL, J. B. & KEEFFE, J. E. 2007c. The impact of vision impairment questionnaire: an assessment of its domain structure using confirmatory factor analysis and rasch analysis. *Investigative Ophthalmology & Visual Science,* 48**,** 1001-6.

LAMOUREUX, E. L., PALLANT, J. F., PESUDOVS, K., TENNANT, A., REES, G., O'CONNOR, P. M. & KEEFFE, J. E. 2008b. Assessing participation in daily living and the effectiveness of rehabiliation in age related macular degeneration patients using the impact of vision impairment scale. *Ophthalmic Epidemiology,* 15**,** 105-13.

LAMOUREUX, E. L., PESUDOVS, K., PALLANT, J. F., REES, G., HASSELL, J. B., CAUDLE, L. E. & KEEFFE, J. E. 2008c. An evaluation of the 10-item vision core measure 1 (VCM1) scale (the Core Module of the Vision-Related Quality of Life scale) using Rasch analysis. *Ophthalmic Epidemiology,* 15**,** 224-33.

LAMOUREUX, E. L., PESUDOVS, K., THUMBOO, J., SAW, S. M. & WONG, T. Y. 2009b. An evaluation of the reliability and validity of the visual functioning questionnaire (VF-11) using Rasch analysis in an Asian population. *Investigative Ophthalmology & Visual Science,* 50**,** 2607-13.

LESKE, D. A., HATT, S. R., LIEBERMANN, L. & HOLMES, J. M. 2012. Evaluation of the Adult Strabismus-20 (AS-20) questionnaire using Rasch analysis. *Investigative Ophthalmology & Visual Science,* 53**,** 2630-9.

LINDER, M., CHANG, T. S., SCOTT, I. U., HAY, D., CHAMBERS, K., SIBLEY, L. M. & WEIS, E. 1999. Validity of the visual function index (VF-14) in patients with retinal disease. *Archives of Ophthalmology,* 117**,** 1611-6.

LLOYD, A. J., LOFTUS, J., TURNER, M., LAI, G. & PLEIL, A. 2013. Psychometric validation of the Visual Function Questionnaire-25 in patients with diabetic macular edema. *Health & Quality of Life Outcomes,* 11**,** 10.

LUNDSTROM, M., FREGELL, G. & SJOBLOM, A. 1994. Vision related daily life problems in patients waiting for cataract extraction. *British Journal of Ophthalmology,* 78**,** 608-611.

LUNDSTROM, M. & PESUDOVS, K. 2009. Catquest-9SF patient outcomes questionnaire: nine-item short-form Rasch-scaled revision of the Catquest questionnaire. *Journal of Cataract & Refractive Surgery,* 35**,** 504-13.

LUNDSTROM, M., ROOS, P., JENSEN, S. & FREGELL, G. 1997. Catquest questionnaire for use in cataract surgery care: description, validity, and reliability. *Journal of Cataract & Refractive Surgery,* 23**,** 1226-36.

MA, S.-L., SHEA, J. A., GALETTA, S. L., JACOBS, D. A., MARKOWITZ, C. E., MAGUIRE, M. G. & BALCER, L. J. 2002. Self-reported visual dysfunction in multiple sclerosis: new data from the VFQ-25 and development of an MS-specific vision questionnaire. *American Journal of Ophthalmology,* 133**,** 686-692.

MANGIONE, C. M., BERRY, S., SPRITZER, K., JANZ, N. K., KLEIN, R., OWSLEY, C. & LEE, P. P. 1998a. Identifying the content area for the 51-item National Eye Institute Visual Function Questionnaire: results from focus groups with visually impaired persons. *Archives of Ophthalmology,* 116**,** 227-33.

MANGIONE, C. M., LEE, P. P., GUTIERREZ, P. R., SPRITZER, K., BERRY, S., HAYS, R. D. & NATIONAL EYE INSTITUTE VISUAL FUNCTION QUESTIONNAIRE FIELD TEST, I. 2001. Development of the 25-item National Eye Institute Visual Function Questionnaire. *Archives of Ophthalmology,* 119**,** 1050-8.

MANGIONE, C. M., LEE, P. P., PITTS, J., GUTIERREZ, P., BERRY, S. & HAYS, R. D. 1998b. Psychometric properties of the National Eye Institute Visual Function Questionnaire (NEI-VFQ). NEI-VFQ Field Test Investigators. *Archives of Ophthalmology,* 116**,** 1496-504.

MANGIONE, C. M., PHILLIPS, R. S., SEDDON, J. M., LAWRENCE, M. G., COOK, E. F., DAILEY, R. & GOLDMAN, L. 1992. Development of the 'Activites of Daily Vision Scale'. *Medical Care,* 30**,** 1111-1126.

MARELLA, M., GOTHWAL, V. K., PESUDOVS, K. & LAMOUREUX, E. 2009. Validation of the visual disability questionnaire (VDQ) in India. *Optometry & Vision Science,* 86**,** E826-35.

MARELLA, M., PESUDOVS, K., KEEFFE, J. E., O'CONNOR, P. M., REES, G. & LAMOUREUX, E. L. 2010. The psychometric validity of the NEI VFQ-25 for use in a low-vision population. *Investigative Ophthalmology & Visual Science,* 51**,** 2878-84.

MASSOF, R. W. 1998. A systems model for low vision rehabilitation. II. Measurement of vision disabilities. *Optometry & Vision Science,* 75**,** 349-73.

MASSOF, R. W. 2007. An interval-scaled scoring algorithm for visual function questionnaires. *Optometry & Vision Science,* 84**,** 689-704.

MASSOF, R. W., AHMADIAN, L., GROVER, L. L., DEREMEIK, J. T., GOLDSTEIN, J. E., RAINEY, C., EPSTEIN, C. & BARNETT, G. D. 2007. The Activity Inventory: an adaptive visual function questionnaire. *Optometry & Vision Science,* 84**,** 763-74.

MASSOF, R. W., HSU, C. T., BAKER, F. H., BARNETT, G. D., PARK, W. L., DEREMEIK, J. T., RAINEY, C. & EPSTEIN, C. 2005a. Visual disability variables. I: the importance and difficulty of activity goals for a sample of low-vision patients. *Archives of Physical Medicine & Rehabilitation,* 86**,** 946-53.

MASSOF, R. W., HSU, C. T., BAKER, F. H., BARNETT, G. D., PARK, W. L., DEREMEIK, J. T., RAINEY, C. & EPSTEIN, C. 2005b. Visual disability variables. II: The difficulty of tasks for a sample of low-vision patients. *Archives of Physical Medicine & Rehabilitation,* 86**,** 954-67.

MCALINDEN, C., PESUDOVS, K. & MOORE, J. E. 2010. The development of an instrument to measure quality of vision: the Quality of Vision (QoV) questionnaire. *Investigative Ophthalmology & Visual Science,* 51**,** 5537-45.

MCALINDEN, C., SKIADARESI, E., GATINEL, D., CABOT, F., HUANG, J. & PESUDOVS, K. 2013. The quality of vision questionnaire: subscale interchangeability. *Optometry & Vision Science,* 90**,** 760-764.

MCALINDEN, C., SKIADARESI, E., MOORE, J. E. & PESUDOVS, K. 2011a. Subscale assessment of the NEI-RQL-42 questionnaire with rasch analysis. *Investigative Ophthalmology & Visual Science,* 52**,** 5685-5694.

MCALINDEN, C., SKIADARESI, E., PESUDOVS, K. & MOORE, J. E. 2011b. Quality of vision after myopic and hyperopic laser-assisted subepithelial keratectomy. *Journal of Cataract & Refractive Surgery,* 37**,** 109-1100.

MENNEM, T. A., WARREN, M. & YUEN, H. K. 2012. Preliminary validation of a vision-dependent activities of daily living instrument on adults with homonymous hemianopia. *American Journal of Occupational Therapy,* 66**,** 478-82.

MILLS, R. P. & DRANCE, S. M. 1986. Esterman disability rating in severe glaucoma. *Ophthalmology,* 93**,** 371-378.

MISAJON, R., HAWTHORNE, G., RICHARDSON, J., BARTON, J., PEACOCK, S., IEZZI, A. & KEEFFE, J. 2005. Vision and quality of life: the development of a utility measure. *Investigative Ophthalmology & Visual Science,* 46**,** 4007-15.

MOGHIMI, S., RIAZI ESFAHANI, M. & MAGHSOUDIPOUR, M. 2007. Visual function after implantation of aniridia intraocular lens for traumatic aniridia in vitrectomized eyes. *European Journal of Ophthalmology,* 17**,** 660-665.

MURTHY, G. V., GUPTA, S. K., THULASIRAJ, R. D., VISWANATH, K., DONOGHUE, E. M. & FLETCHER, A. E. 2005. The development of the Indian vision function questionnaire: questionnaire content. *British Journal of Ophthalmology,* 89**,** 498-503.

NAIK, R. K., RENTZ, A. M., FOSTER, C. & ET AL. 2013. Normative comparison of patient-reported outcomes in patients with noninfectious uveitis. *JAMA Ophthalmology,* 131**,** 219-225.

NELSON, P., ASPINALL, P. & O'BRIEN, C. 1999. Patients' perception of visual impairment in glaucoma: a pilot study. *British Journal of Ophthalmology,* 83**,** 546-52.

ORR, P., RENTZ, A. M., MARGOLIS, M. K., REVICKI, D. A., DOLAN, C. M., COLMAN, S., FINE, J. T. & BRESSLER, N. M. 2011. Validation of the National Eye Institute Visual Function Questionnaire-25 (NEI VFQ-25) in age-related macular degeneration. *Investigative Ophthalmology & Visual Science,* 52**,** 3354-9.

PAGER, C. K. 2004. Assessment of visual satisfaction and function after cataract surgery. *Journal of Cataract & Refractive Surgery,* 30**,** 2510-6.

PESUDOVS, K., CAUDLE, L. E., REES, G. & LAMOUREUX, E. L. 2008. Validity of a visual impairment questionnaire in measuring cataract surgery outcomes. *Journal of Cataract & Refractive Surgery,* 34**,** 925-33.

PESUDOVS, K. & COSTER, D. J. 1998. An instrument for assessment of subjective visual disability in cataract patients. *British Journal of Ophthalmology,* 82**,** 617-24.

PESUDOVS, K., GARAMENDI, E., KEEVES, J. P. & ELLIOTT, D. B. 2003. The Activities of Daily Vision Scale for cataract surgery outcomes: re-evaluating validity with Rasch analysis. *Investigative Ophthalmology & Visual Science,* 44**,** 2892-9.

PESUDOVS, K., GOTHWAL, V. K., WRIGHT, T. & LAMOUREUX, E. L. 2010a. Remediating serious flaws in the National Eye Institute Visual Function Questionnaire. *Journal of Cataract & Refractive Surgery,* 36**,** 718-32.

PESUDOVS, K., WRIGHT, T. A. & GOTHWAL, V. K. 2010b. Visual disability assessment: valid measurement of activity limitation and mobility in cataract patients. *British Journal of Ophthalmology,* 94**,** 777-81.

PIERMAROCCHI, S., VARANO, M., PARRAVANO, M., ODDONE, F., SARTORE, M., FERRARA, R., SERA, F. & VIRGILI, G. 2011. Quality of Vision Index: a new method to appraise visual function changes in age-related macular degeneration. *European Journal of Ophthalmology,* 21**,** 55-66.

PRAGER, T. C., CHUANG, A. Z., SLATER, C. H., GLASSER, J. H. & RUIZ, R. S. 2000. The Houston Vision Assessment Test (HVAT): an assessment of validity. The Cataract Outcome Study Group. *Ophthalmic Epidemiology,* 7**,** 87-102.

RAPHAEL, B. A., GALETTA, K. M., JACOBS, D. A., MARKOWITZ, C. E., LIU, G. T., NANO-SCHIAVI, M. L., GALETTA, S. L., MAGUIRE, M. G., MANGIONE, C. M., GLOBE, D. R. & BALCER, L. J. 2006. Validation and test characteristics of a 10-item neuro-ophthalmic supplement to the NEI-VFQ-25. *American Journal of Ophthalmology,* 142**,** 1026-35.

ROWE, F. J., WRIGHT, D., BRAND, D., JACKSON, C., HARRISON, S., MAAN, T., SCOTT, C., VOGWELL, L., PEEL, S., AKERMAN, N., DODRIDGE, C., HOWARD, C., SHIPMAN, T., SPERRING, U., MACDIARMID, S. & FREEMAN, C. 2013. A prospective profile of visual field loss following stroke: Prevalence, type, rehabilitation and outcome. *BioMed Research International* [Online], 2013. Available: <http://dx.doi.org/10.1155/2013/719096>.

RYAN, B., COURT, H. & MARGRAIN, T. H. 2008. Measuring low vision service outcomes: Rasch analysis of the seven-item National Eye Institute Visual Function Questionnaire. *Optometry & Vision Science,* 85**,** 112-21.

SCHMIER, J. K., HALPERN, M. T. & COVERT, D. 2006. Validation of the Daily Living Tasks Dependent on Vision (DLTV) questionnaire in a U.S. population with age-related macular degeneration. *Ophthalmic Epidemiology,* 13**,** 137-43.

SLOANE, M. E., BALL, K., OWSLEY, C., BRUNI, J. R. & ROENKER, D. L. The visual activities questionnaire: Developing an instrument for assing problems in everyday visual tasks. Technical Digest, Noninvasive assessment of the visual system, Topical meeting of the Optical Society of America, 1992 Sante Fe, New Mexico. Optical Society of America.

STEINBERG, E. P., TIELSCH, J. M., SCHEIN, O. D., JAVITT, J. C., SHARKEY, P., CASSARD, S. D., LEGRO, M. W., DIENER-WEST, M., BASS, E. B., DAMIANO, A. M. & ET AL. 1994. The VF-14. An index of functional impairment in patients with cataract. *Archives of Ophthalmology,* 112**,** 630-8.

STELMACK, J., SZLYK, J. P., STELMACK, T., BABCOCK-PARZIALE, J., DEMERS-TURCO, P., WILLIAMS, R. T. & MASSOF, R. W. 2004a. Use of Rasch person-item map in exploratory data analysis: a clinical perspective. *Journal of Rehabilitation Research & Development,* 41**,** 233-41.

STELMACK, J. A. & MASSOF, R. W. 2007. Using the VA LV VFQ-48 and LV VFQ-20 in low vision rehabilitation. *Optometry & Vision Science,* 84**,** 705-9.

STELMACK, J. A., SZLYK, J. P., STELMACK, T. R., DEMERS-TURCO, P., WILLIAMS, R. T., MORAN, D. & MASSOF, R. W. 2004b. Psychometric properties of the Veterans Affairs Low-Vision Visual Functioning Questionnaire. *Investigative Ophthalmology & Visual Science,* 45**,** 3919-28.

STELMACK, J. A., SZLYK, J. P., STELMACK, T. R., DEMERS-TURCO, P., WILLIAMS, R. T., MORAN, D. & MASSOF, R. W. 2006. Measuring outcomes of vision rehabilitation with the veterans affairs low vision visual functioning questionnaire. *Investigative Ophthalmology & Visual Science,* 47**,** 3253-3261.

SZLYK, J. P., STELMACK, J., MASSOF, R. W., STELMACK, T. R., DEMERS-TURCO, P., WILLIAMS, R. T. & WRIGHT, B. D. 2004. Performance of the veterans affairs low vision vision visual functioning questionnaire. *Journal of Visual Impairment and Blindness,* 98**,** 261-275.

TURANO, K. A., GERUSCHAT, D. R., STAHL, J. W. & MASSOF, R. W. 1999. Perceived visual ability for independent mobility in persons with retinitis pigmentosa. *Investigative Ophthalmology & Visual Science,* 40**,** 865-77.

TURANO, K. A., MASSOF, R. W. & QUIGLEY, H. A. 2002. A self-assessment instrument designed for measuring independent mobility in RP patients: generalizability to glaucoma patients. *Investigative Ophthalmology & Visual Science,* 43**,** 2874-81.

UUSITALO, R. J., BRANS, T., PESSI, T. & TARKKANEN, A. 1999. Evaluating cataract surgery gains by assessing patients' quality of life using the VF-7. *Journal of Cataract & Refractive Surgery,* 25**,** 989-94.

VALBUENA, M., BANDEEN-ROCHE, K., RUBIN, G. S., MUNOZ, B. & WEST, S. K. 1999. Self-reported assessment of visual function in a population-based study: the SEE project. Salisbury Eye Evaluation. *Investigative Ophthalmology & Visual Science,* 40**,** 280-8.

VAN DE GRAAF, E. S., VAN DER STERRE, G. W., POLLING, J. R., VAN KEMPEN, H., SIMONSZ, B. & SIMONSZ, H. J. 2004. Amblyopia & Strabismus Questionnaire: design and initial validation. *Strabismus,* 12**,** 181-93.

VELOZO, C. A., WARREN, M., HICKS, E. & BERGER, K. A. 2013. Generating clinical outputs for self-reports of visual functioning. *Optometry & Vision Science,* 90**,** 765-75.

VIANYA-ESTOPA, M., ELLIOTT, D. B. & BARRETT, B. T. 2010. An evaluation of the Amblyopia and Strabismus Questionnaire using Rasch analysis. *Investigative Ophthalmology & Visual Science,* 51**,** 2496-503.

VISWANATHAN, A. C., MCNAUGHT, A. I., POINOOSAWMY, D., FONTANA, L., CRABB, D. P., FITZKE, F. W. & HITCHINGS, R. A. 1999. Severity and stability of glaucoma. *Archives of Ophthalmology,* 117.

WANG, B., AUNG, T., MARELLA, M., ZHENG, Y., WONG, T. Y., PERERA, S., WONG, T. T., HO, C. L. & LAMOUREUX, E. L. 2013. Impact of bilateral open and closed-angle glaucoma on glaucoma-specific functioning in Asians. *Journal of Glaucoma,* 22**,** 330-5.

WEIH, L. M., HASSELL, J. B. & KEEFFE, J. 2002. Assessment of the impact of vision impairment. *Investigative Ophthalmology & Visual Science,* 43**,** 927-35.

WOLFFSOHN, J. S. & COCHRANE, A. L. 2000. Design of the low vision quality-of-life questionnaire (LVQOL) and measuring the outcome of low-vision rehabilitation. *American Journal of Ophthalmology,* 130**,** 793-802.

WOLFFSOHN, J. S., COCHRANE, A. L. & WATT, N. A. 2000. Implementation methods for vision related quality of life questionnaires. *British Journal of Ophthalmology,* 84**,** 1035-1040.
